# Perturb-Multimodal: a platform for pooled genetic screens with sequencing and imaging in intact mammalian tissue

**DOI:** 10.1016/j.cell.2025.05.022

**Published:** 2025-06-12

**Authors:** Reuben A. Saunders, William E. Allen, Xingjie Pan, Jaspreet Sandhu, Jiaqi Lu, Thomas K. Lau, Karina Smolyar, Zuri A. Sullivan, Catherine Dulac, Jonathan S. Weissman, Xiaowei Zhuang

**Affiliations:** 1 Howard Hughes Medical Institute, Chevy Chase, MD 20815, USA; 2 Whitehead Institute, Cambridge, MA 02139, USA; 3 University of California, San Francisco, San Francisco, CA 94158, USA; 4 Society of Fellows, Harvard University, Cambridge, MA 02138, USA; 5 Department of Chemistry and Chemical Biology and Department of Physics, Harvard University, Cambridge, MA 02138, USA; 6 Division of Gastroenterology, Massachusetts General Hospital, Boston, MA 02114, USA; 7 Department of Statistics, Stanford University, Stanford, CA 94305; 8 Department of Biology, Massachusetts Institute of Technology, Cambridge, MA 02139 USA; 9 Department of Molecular and Cellular Biology, Harvard University, Cambridge, MA 02138, USA; 10 Present address: Society of Fellows, Harvard University, MA 02138, USA; 11 Present address: Department of Developmental Biology, Stanford University School of Medicine, Stanford, CA 94305

## Abstract

**SUMMARY:**

Metazoan life requires coordinated activities of thousands of genes in spatially organized cell types. Understanding the basis of tissue function requires approaches to dissect the genetic control of diverse cellular and tissue phenotypes *in vivo*. Here, we present Perturb-Multimodal (Perturb-Multi), a paired imaging and sequencing method to construct large-scale, multimodal genotype-phenotype maps in tissues with pooled genetic perturbations. Using imaging, we identify perturbations in individual cells while simultaneously measuring their gene-expression profiles and subcellular morphology. Using single-cell sequencing, we measure full transcriptomic responses to the same perturbations. We apply Perturb-Multi to study hundreds of genetic perturbations in mouse liver. We reveal candidate genetic regulators and mechanisms underlying the dynamic control of hepatocyte zonation, the unfolded protein response, and steatosis. Perturb-Multi accelerates discoveries of the genetic basis of complex cell and tissue physiology and provides critical training data for emerging machine learning models of cellular function.

**In Brief:**

Pairing imaging and sequencing to simultaneously measure gene expression and morphological phenotypes in diverse genetically perturbed cells in mosaic animals, Perturb-Multimodal enables systematic exploration of gene function in intact tissues and provides insights into genetic regulators and dynamic control of liver functions.

## Graphical Abstract

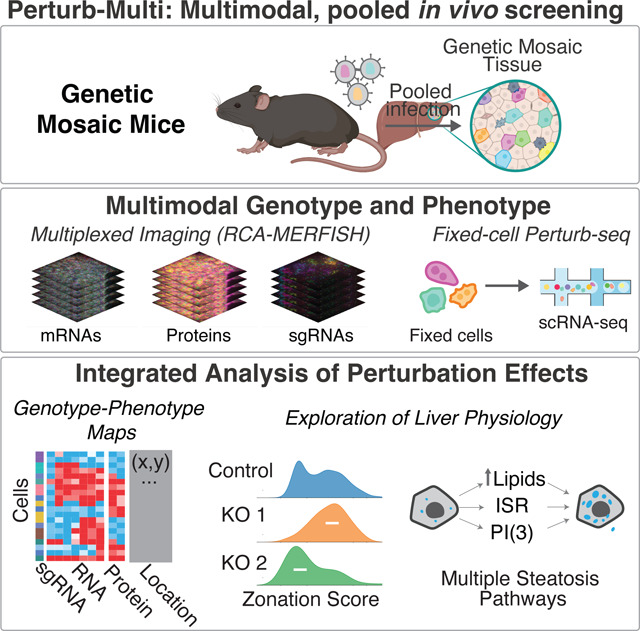

## INTRODUCTION

A central goal of metazoan biology is to understand how the coordinated activities of thousands of genes in diverse cell types produce the physiological functions of tissues. Large-scale atlasing efforts have profiled myriad cell types in normal and disease states^1–3^ and mapped their spatial organization in tissues and organs^4–9^. Nonetheless, causally determining how genes and pathways regulate the states and functions of cells and multicellular networks remains a major challenge. Meeting this challenge requires functional genomics approaches that systematically dissect gene control of cellular and tissue phenotypes in living animals.

Cellular and tissue states manifest across multiple dimensions, from gene expression and subcellular architecture to the organization of cells in tissue neighborhoods^10^. Imaging provides a highly interpretable approach for studying cellular and tissue state, revealing how structural changes reflect normal physiology and disease. Machine learning is transforming quantitative analysis of cellular and tissue morphology at scale, providing unsupervised feature extraction from high-dimensional imaging data^11^. Combined with next-generation sequencing, these approaches promise to offer holistic views of cellular state—linking subcellular structure to gene expression—while preserving spatial context.

Integration of these imaging or sequencing tools for deep cell phenotyping with pooled genetic perturbations has expanded our ability to dissect the genetic bases of biological processes. In cultured cells, Perturb-seq—combining pooled genetic screens with single-cell RNA-seq readouts—has enabled dissection of core cellular processes like the unfolded protein response (UPR)^12^, hematopoiesis^13,14^, and alternative polyadenylation^15^, as well as unsupervised classification of gene function at genome-wide scale^16^. In parallel, imaging-based pooled screening approaches have been developed, employing multiplexed FISH or in situ sequencing to identify perturbations and map them to complex morphological phenotypes with subcellular resolution^17–23^.

Many aspects of physiology demand *in vivo* studies in intact organs. Mosaic animals, where individual cells carry distinct perturbations, can be generated by viral transduction^24,25^, and with Perturb-seq, have enabled profiling of disease-relevant genes in brain^26–28^, skin^29^, and the immune system^13,30^. However, adoption of *in vivo* Perturb-seq has been constrained by challenges in isolating perturbed cells while preserving their integrity^31^. Such approaches also lose spatial and morphological information essential for understanding tissue function.

A comprehensive understanding of how genes control physiological functions will require an integrated approach that links transcriptionally defined cellular states to morphological and functional phenotypes. Imaging-based pooled screening approaches have recently been expanded to include multimodal protein and RNA measurements in cultured cells or xenograft models^19,32–34^. However, new approaches are needed to connect transcriptome-wide expression profiling with structural and functional phenotyping and causally map diverse genetic perturbations to phenotypes within native tissues.

Here, we report the development of Perturb-Multimodal (Perturb-Multi), which enables pooled genetic screening in native tissue with rich, multimodal phenotypic readouts. We establish methods for Perturb-seq and imaging-based pooled screening in fixed tissue, enabling joint sequencing and imaging analysis of diverse perturbations in the same tissue. Our approach allows genome-wide transcriptional analysis of each perturbed cell by scRNA-seq, as well as multiplexed protein and RNA imaging, using immunofluorescence and *in situ* amplification followed by multiplexed error-robust FISH (MERFISH)^35^. We apply Perturb-Multi to the mouse liver to examine how hundreds of gene perturbations affect transcriptional state, subcellular morphology, and tissue organization. By integrating these data, we explore genes and principles that enable dynamic regulation of hepatocyte zonation, reveal broad effects of proteostatic stress on secreted proteins, and uncover distinct pathways leading to steatosis. Beyond dissecting the genetic basis of complex physiology, Perturb-Multi provides training data for emerging machine learning efforts to create predictive models of cells and tissues^36–38^.

## RESULTS

### Multimodal *in vivo* pooled genetic screens through imaging and sequencing

We aimed to profile the effects of genetic perturbations with multimodal cell-state characterization at subcellular resolution across distinct cell types in living mouse tissue. Examining complex, multimodal phenotypes would allow exploration of a wider range of cellular physiology and gene function than previous pooled genetic screens in the liver^25,39–41^, which were primarily focused on cell survival or proliferation.

**Figure 1:**
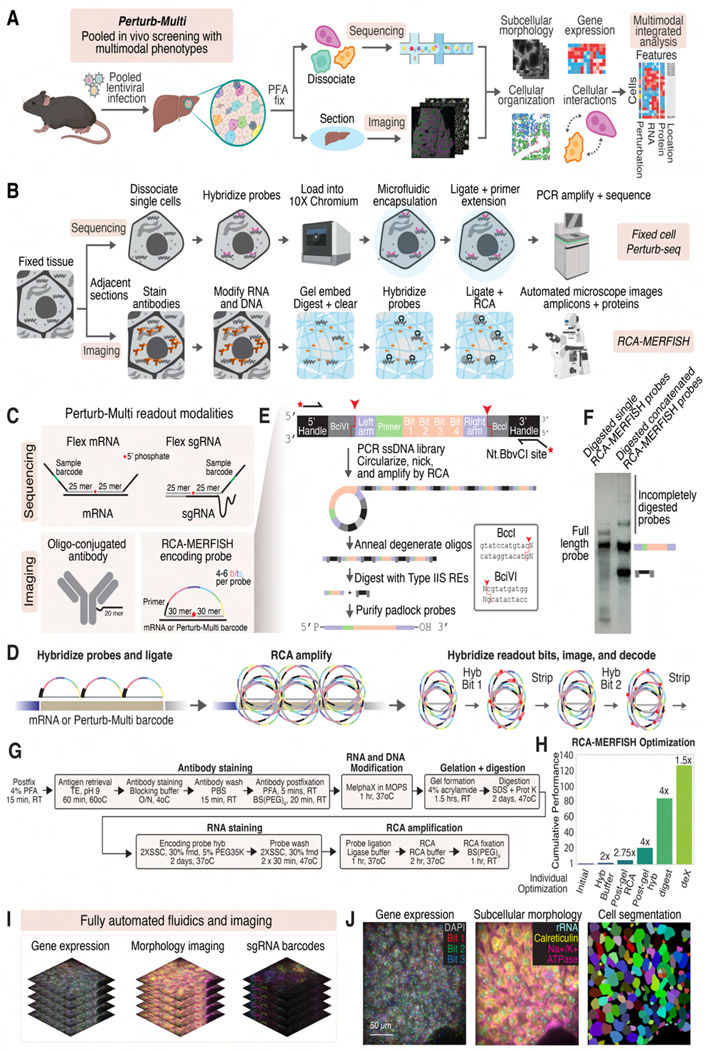
Perturb-Multimodal (Perturb-Multi): Pooled *in vivo* multimodal genetic screens through imaging and sequencing A. Lentiviral delivery of genetic perturbations into liver tissue of living mouse, followed by tissue fixation and parallel sequencing and imaging measurements for integrated genotype-phenotype analysis. B. Dual readout methods: (Top) Perturb-Multi sequencing – dissociated cells from fixed tissues sections analyzed via hybridization-based Perturb-seq using 10x Flex platform; (Bottom) Perturb-Multi imaging – adjacent tissue sections labeled with oligo-conjugated antibodies and embedded in polyacrylamide with RNA and oligo-antibodies anchored, followed by clearing and detection with RCA-MERFISH. C. The four readout modalities: 10x Flex split probes for mRNA; custom split probes for sgRNAs; acrydite-oligo antibodies for multiplex immunofluorescence of proteins; RCA-MERFISH padlock probes for mRNA and perturbation barcodes. Each padlock probe contains multiple readout sequences (colored) encoding the target RNA (code reading “1” at the bits corresponding to the present readout sequences and “0” at the other bits) D. RCA-MERFISH workflow: RNAs are hybridized with padlock encoding probes, which are then ligated, rolling circle amplified, and detected by multiple rounds of hybridization with fluorescent readout probes complementary to the readout sequence. E. Synthesis of full-length, padlock encoding probes from array-synthesized oligo pools via PCR amplification, circularization, nicking, RCA, and Type IIS restriction digestion (see STAR Methods). F. Denaturing PAGE analysis comparing oligonucleotide standard (left) and RCA-MERFISH encoding probe library (right). G. The optimized sample-preparation protocol for Perturb-Multi imaging. H. Cumulative RCA-MERFISH performance improvements after protocol optimization including hybridization crowding-agent improvement, post-gel padlock probe hybridization and RCA, in-gel digestion, and decrosslinking refinement. I. Multimodal readout of RNA and protein through RCA-MERFISH (for mRNAs of 209 genes and 456 sgRNAs) and sequential rounds of immunofluorescence and FISH (for 14 proteins and 4 abundant RNAs), with automated fluidics and imaging. J. Multimodal measurement of gene expression (left, first three bits of 18-bit MERFISH mRNA imaging shown) and subcellular morphology (center; four of the 14 protein and 4 abundant targets shown), with machine learning-based segmentation of cells (right). See also Figures S1, S2 and Table S1.

Building on established approaches for *in vivo* CRISPR library delivery^25^, we infected hepatocytes in Cas9 transgenic mice^42^ with a lentiviruses pool, each carrying a distinct CRISPR guide and RNA barcode (Figure 1A). After perfusion fixation, we interrogated the tissue with Perturb-Multi for paired single-cell sequencing and imaging readouts (Figure 1A). scRNA-seq provides genome-wide transcriptional profiles, while imaging captures subcellular morphologies, protein and mRNA distributions, and spatial organization of cells. Linking these phenotype measurements via shared perturbation identities reveals multiple facets of cell and gene function.

The development of Perturb-Multi required extensive technological innovation in both imaging and single-cell sequencing (Figure 1B). For imaging, we aimed for rapid, simultaneous, highly-multiplexed readout of perturbation barcodes, endogenous mRNAs, and proteins in morphology-preserved tissues (Figure 1B, bottom). To image short perturbation barcodes, we developed an RCA-MERFISH protocol where RNAs are detected through rolling circle amplification (RCA)^43–45^ of padlock probes that bind to target RNAs and harbor multiple readout sequences, forming a MERFISH barcodes that can be detected with sequential rounds of isothermal hybridization without enzymatic steps in an automated flow cell (Figures 1C, bottom right, and 1D). We further developed a strategy for the cost-effective generation of full-length padlock probes (Figure 1E, 1F). Because RCA degrades linear MERFISH encoding probes^35^ (Figure S1A-S1C), we used RCA-MERFISH for detecting both perturbation barcodes and endogenous mRNAs, with RCA-compatible crowding agents (Figure S1D-S1F).

For multimodal RNA and protein imaging, perfusion-fixation provides superior preservation of cell and tissue morphology^46^ over fresh-frozen samples but inhibits RCA (Figure S1G). We addressed this by using polyacrylamide gel to anchor RNAs, allowing tissue clearing^47^ and in-gel probe hybridization and RCA (Figures S1G-S1H). Proteins were imaged with oligo-conjugated antibodies^48–50^ co-embedded in the gel (Figures 1C, Bottom left) with decrosslinking conditions optimized for protein and RNA co-detection (Figure S1J, S1K). Collectively, our optimized imaging protocol (Figures 1B, bottom, 1G; STAR Methods) improved RCA-MERFISH detection efficiency by >100X (Figures 1H–1J and S1L-S1N).

To enable transcriptome-wide measurements in the same fixed tissues, we developed a fixed-cell Perturb-seq approach. We built upon the 10x Flex platform, which uses microfluidic encapsulation of cells pre-hybridized to a library of split probes for mRNA detection, and developed custom split probes targeting sgRNAs to measure perturbations (STAR Methods, Figures 1B, top and 1C, top). This allowed imaging and sequencing on adjacent sections, minimizing animal-to-animal variability and enabling multimodal analyses with internal controls (Figures 1B). Moreover, our approach dramatically simplifies the *in vivo* Perturb-seq workflow due to the stability of fixed tissue through harvesting, cryopreservation, dissociation, flow sorting, and single-cell library preparation steps in comparison to live cells or isolated nuclei^31,51^.

### Multimodal analyses of cell-type and cell-state organization in the unperturbed liver

**Figure 2:**
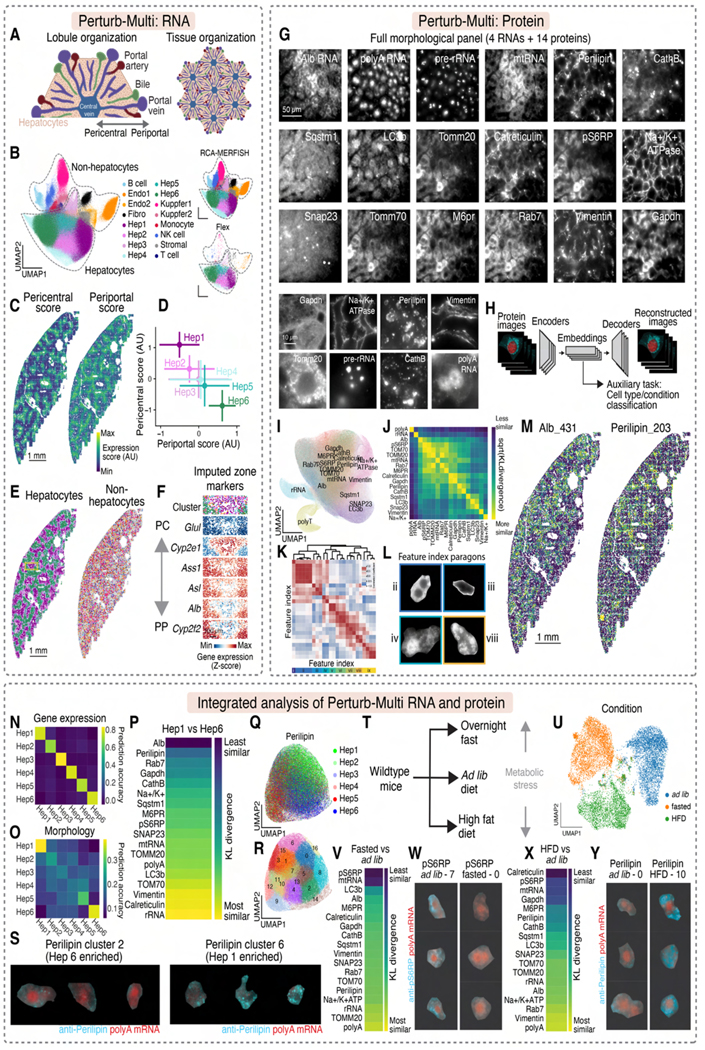
Heterogeneity in transcription and subcellular morphology by cell type and state A. Liver lobule organization showing pericentral to periportal axis (BioRender). B. Integrated UMAP of RCA-MERFISH and Flex scRNA-seq data (Left: both; Top right: RCA-MERFISH; Bottom right: Flex) showing liver cell types, as determined by unsupervised clustering. C. Spatial distribution of periportal (left) and pericentral (right) scores in hepatocytes based on marker gene expression (STAR Methods). D. Periportal vs pericentral gene expression scores across hepatocyte subtypes. E. Spatial organization of hepatocyte subtypes (left) and non-hepatocyte cells (right). F. Spatial map of hepatocyte zone marker expression radially organized around a central vein. Yellow-boxed region from (E) with cell types (top) and imputed gene expression are separately scaled for each gene (bottom). PP: Periportal; PC: Pericentral. G. Morphology panel showing 4 abundant RNA species and 14 proteins (top) with zoomed details of a subset of targets (bottom). H. Deep learning autoencoder diagram reducing protein morphologies to 512-dimensional embeddings using the VQ-VAE model with auxiliary tasks of discriminating cell types, cell states, or conditions. I. UMAP of subcellular morphology image embeddings colored by channel (target protein and abundant RNA) identity. J. Similarity of subcellular morphology channel embedding quantified by Kullback-Leibler (KL) divergence. K. Correlation heatmap of high-signal features across image embeddings, ordered by hierarchical clustering to reveal nine feature classes (see Figure S5C). L. Cells displaying high weight scores from selected feature classes, including (ii) double nucleus, (iii) membrane enrichment, (iv) diffuse expression, and (viii) punctate patterns. M. Tissue-scale spatial organization of morphological embedding features. Left: *Albumin* mRNA feature 431; Right: Perilipin feature 203 N. Confusion matrix of hepatocyte subtype classification accuracy on held-out cells using MERFISH transcriptomic data of 209 genes. O. Confusion matrix of hepatocyte subtype classification accuracy on held-out cells using morphological feature embeddings from 14 proteins and 4 abundant RNAs. P. Heatmap of mutual information between hepatocyte subtypes Hep1 and Hep6 for individual morphological channels, quantified by quantified by KL divergence. Q. UMAP of anti-Perilipin morphological embeddings of single-cell images, colored by hepatocyte subtype. R. UMAP of anti-Perilipin morphological embeddings of single-cell images, colored by Leiden cluster. S. Sampling of hepatocytes from Perilipin embedding clusters 2 (Hep 6-enriched) and 6 (Hep 1-enriched). T. Diet experiment diagram. U. scRNA-seq UMAP from mice under ad lib, overnight fasting, or high-fat diet (HFD) conditions. V. Heatmap of mutual information between *ad lib* and fasted hepatocytes for individual morphological embedding features, quantified by quantified by KL divergence. W. Sampling of hepatocytes from anti-p-S6 RP embedding cluster 7 (from *ad lib* condition) and cluster 0 (from fasted condition). Cluster 7 is most enriched in the *ad lib* condition and cluster 0 is most enriched in the fasted condition. X. Same as (V), but for morphological channel embeddings between *ad lib* and HFD hepatocytes. Y. Same as (W) but for anti-perilipin embedding cluster 0 (from *ad lib* condition) and cluster 10 (from HFD condition). See also Figures S2-S6 and Tables S2-S4.

We first applied Perturb-Multi phenotype measurements to wild-type mouse livers under different physiological conditions to examine the spatial organization of cell types and states. A fundamental model in cell biology^52–55^, hepatocytes – the principal cell type of the liver – possess a well-defined molecular composition^56,57^ and are readily infected by viruses for perturbation delivery. Anatomically, the liver comprises radially arranged periportal and pericentral zones centered around respective veins, repeated throughout the tissue (Figure 2A)^58^. Periportal hepatocytes specialize in oxidative metabolism, gluconeogenesis, and the urea cycle, while pericentral hepatocytes specialize in glycolysis and lipogenesis^58^.

Using RCA-MERFISH, we measured endogenous mRNAs for 209 genes, including cell type markers and genes important for liver function (Tables S1, Figure S1M, S1N). The high-contrast zonal patterns of markers indicate a high specificity of RCA-MERFISH detection (Figure S2). We performed Flex-based scRNA-seq on adjacent sections and integrated RCA-MERFISH and scRNA-seq data. Unsupervised clustering revealed multiple hepatocyte subtypes and non-hepatocyte cell types (Figure 2B, Figure S3). Calculations of the periportal and pericentral scores with known markers revealed a continuous gradient of hepatocytes within the lobule, whereas non-hepatocytes exhibited more random spatial distributions (Figures 2C-2E, S3B). Full-transcriptome imputation of the imaged cells recapitulated known zonated expression of many genes (Figure 2F, cf. ^59^).

To examine morphological states, we designed an 18-target panel comprising protein markers of subcellular structures and signaling pathways, plus four abundant RNAs (Figure 2G; Tables S2-S4). To analyze the imaging data, we used deep learning for dimensionality reduction^60,61^ and feature extraction at the single-cell level. For each segmented cell, we reduced each of the morphological images to a 512-dimensional embedding (each dimension representing a feature) by training a VQ-VAE network^11^ constrained by an auxiliary task that promoted discrimination of biological variations (e.g. cell types and physiological conditions) (Figures 2H, S4-S5). Morphologically similar imaging targets clustered together in the embedding space (Figure 2I) and exhibited high mutual information (Figure 2J). Individual features often reflected interpretable subcellular distributions (Figures 2K, 2L; Figure S5A-S5D), and many features exhibited zonal patterns across the liver (Figure 2M, S5E).

To compare morphological and transcriptional data, we trained classifiers to predict hepatocyte subtypes using either MERFISH gene-expression profiles or morphological embeddings. Gene-expression profiles distinguished all subtypes (Figure 2N), whereas morphological embeddings only separated the most periportal (Hep6) and pericentral (Hep1) cells (Figure 2O), suggesting that intermediate subtypes may form a morphological continuum or be morphologically heterogeneous.

We identified morphological features that best distinguish the Hep1 and Hep6 states (Figure 2P). mRNA of *Albumin*, an abundant plasma protein gene, and anti-Perilipin, a lipid droplet marker, showed the greatest divergence (Figure 2P), consistent with zonated *Albumin* expression^59^ and lipid metabolism^58^. Perilipin embeddings exhibited subtype-specific patterns with Hep1 cells preferentially showing more lipid droplets than Hep6 cells (Figures 2Q-2S).

We next examined hepatocyte state changes under metabolic stress by fasting mice or feeding them a high-fat diet (HFD), followed by scRNA-seq and imaging (Figure 2T; STAR Methods). These interventions induced large gene expression changes (Figure 2U, Figure S6A). Under *ad libitum* conditions, many genes varied along the periportal–pericentral axis (Figure S6B). Notably, fasting and HFD caused gene-expression changes with markedly different spatial characteristics. Fasting upregulated lipid biosynthesis genes in pericentral cells relative to periportal cells, whereas HFD led to the opposite trend (Figure S6C-S6E), suggesting an interplay among fasting/satiety signaling, lipid-biosynthesis regulation, and hepatocyte zonation.

We identified imaging targets whose feature embeddings best distinguished the physiological conditions. Phospho-S6 ribosomal protein (pS6RP), an mTOR marker, most clearly distinguished fasted from *ad libitum* conditions (Figures 2V, 2W), consistent with mTOR-driven phosphorylation under nutrient-rich conditions^55,62^. Perilipin was altered between *ad libitum* and HFD conditions (Figures 2X, 2Y), consistent with lipid-droplet accumulation in HFD^63^. Calreticulin and pS6RP also changed with HFD, potentially due to exclusion by lipid droplets (Figures S6F, S6G).

Our paired sequencing and imaging experiments generated an integrated map of liver transcription and subcellular morphology, linking morphological heterogeneity to transcriptionally defined cell types, their spatial organization, and the animal’s physiological state and highlighting complementary insights provided by the multimodal readouts.

### Large-scale *in vivo* multimodal screening in CRISPR mosaic livers

Next, we combined multimodal phenotyping with pooled *in vivo* CRISPR screening to investigate the genetic basis of liver functions. We targeted 202 genes (each with two sgRNAs) plus 50 negative controls. The perturbed genes (Table S5) are involved in diverse aspects of liver physiology, including metabolism, signaling, transcription, translation, and protein trafficking. We also targeted genes with high or specific expression in hepatocytes, including some of uncertain function.

**Figure 3:**
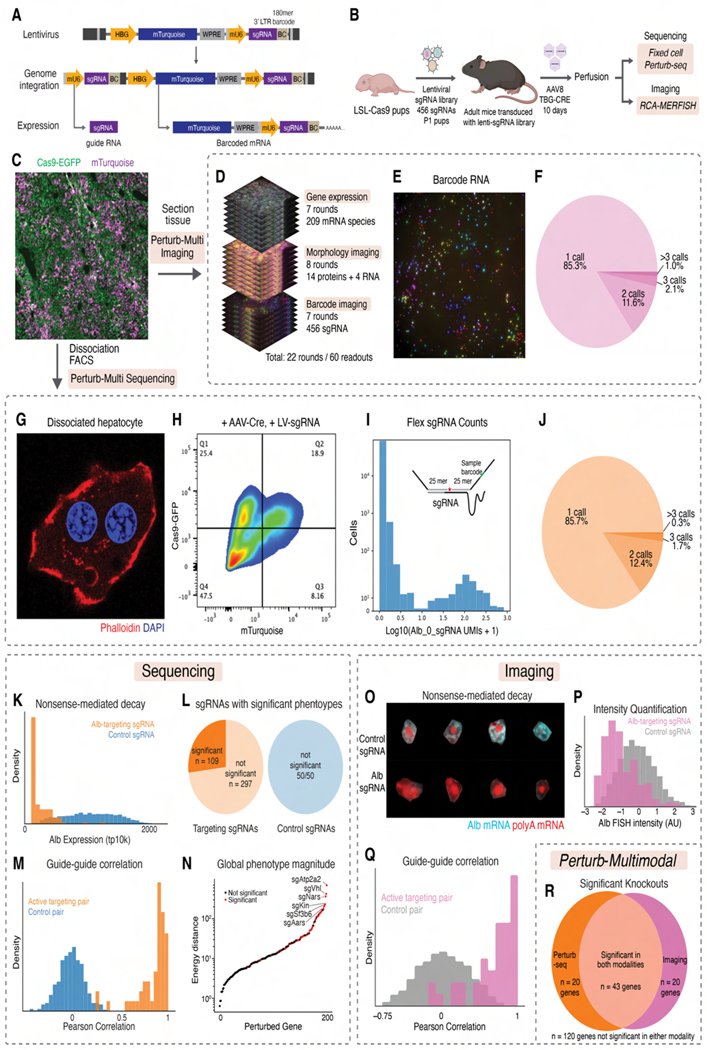
Large-scale, multimodal *in vivo* screening in CRISPR mosaic livers A. Lentiviral CROP-seq vector for dual-mode mosaic screens with mU6-driven sgRNA expression and hepatocyte promoter driving expression of mTurquoise transcripts with perturbation-specific barcode in the 3’ UTR. B. CRISPR experiment workflow: LSL-Cas9 pups was injected with sgRNA library, followed by Cas9 activation in young adults via AAV8 TBG-CRE and perfusion-fixation of livers for RCA-MERFISH or Perturb-seq. C. Fluorescence micrograph of PFA-perfused, lentivirus- and AAV-transduced liver tissue showing Cas9-EGFP (green) and sgRNA-mTurquoise (purple) expression. D. Multimodal readout of 209 endogenous mRNAs and 456 perturbation barcodes via RCA-MERFISH and 14 proteins and 4 abundant RNAs via sequential imaging. E. Representative fluorescence micrograph showing the first three (of 21 total) bits of RCA-MERFISH perturbation imaging. F. Distribution of barcode calls per sgRNA-harboring cell: 85.3% with one barcode, 14.7% with two or more. Only single-barcode cells were analyzed. G. Fluorescence micrograph of a hepatocyte dissociated from fixed liver (Blue: DAPI ; Red: phalloidin). H. Flow cytometry of dissociated, PFA-perfused, lentivirus- and AAV-transduced liver tissue and mTurquoise+ and GFP+ cells are selected to enrich for cell containing sgRNA and active Cas9. I. Histogram of Alb_0 sgRNA counts per cell. J. Barcode calls per sgRNA-harboring cell in Perturb-seq: 85.7% with one barcode, 14.3% with two or more. K. *Albumin* mRNA expression histograms comparing cells receiving control vs. *Albumin*-targeting sgRNAs in Perturb-seq data. L. Fraction of sgRNAs causing significant Perturb-seq phenotypes: 109/406 targeting sgRNAs (27%) vs. 0/50 non-targeting sgRNAs (0%) by Holm-Šídák-corrected energy distance test (p<0.05). M. Histogram of Pearson correlations of pseudobulk Perturb-seq phenotypes between active sgRNA pairs targeting same gene, versus control sgRNA pairs. N. Knockouts ranked by energy distance between cells that received active targeting sgRNA vs cells that received control sgRNA. Energy distance is calculated using the top 20 PCs of Z-normalized Perturb-seq gene expression. O. Unbiased sampling of cells with control sgRNAs and sgRNAs targeting *Albumin* showing *Albumin* mRNA and polyA signals.. P. Histogram comparing *Albumin* mRNA signal between cells receiving control and *Albumin*-targeting sgRNAs, from the imaging data. Q. Histogram of Pearson correlations of pseudobulk imaging intensity phenotypes between active sgRNA pairs targeting same gene, versus control sgRNA pairs. R. Venn diagram of genes with significant knockout effects in imaging and sequencing phenotypes. Phenotype significance is measured by a Holm-Šídák-corrected energy distance permutation tests (p < 0.05). There is significant overlap in the two sets of genes (hypergeometric p < 10^−13^). See also Figure S7 and Tables S1-S6.

We engineered a CROP-seq^64^ lentiviral library expressing mTurquoise and a 185-mer RNA barcode linked to an sgRNA (Figure 3A). The vector was introduced into Cas9-GFP mice at P1^25,65^ via temporal vein injection (Figure 3B). Cas9 expression was induced with AAV-Cre. Ten days post-induction, livers were perfusion-fixed and cryopreserved, showing mosaic mTurquoise alongside uniform GFP expression in hepatocytes (Figure 3C).

For Perturb-Multi imaging, we additionally included padlock probes targeting perturbation-specific barcode sequences, imaged alongside endogenous mRNAs and proteins (Figure 3D and 3E). Most cells contained no perturbation, with perturbed cells typically having a single perturbation, consistent with Poisson-distributed transduction at low MOI (Figure 3F). ~79,000 cells with single perturbations were subsequently analyzed.

For Perturb-Multi sequencing on adjacent sections, we performed fixed-cell Perturb-seq on FACS-enriched mTurquoise+/GFP+ cells (Figure 3G, 3H) with custom split probes targeting sgRNAs (Table S6). Perturbations were called based on UMI count distributions (Figure 3I). Most cells harbored single perturbations (Figure 3J). ~55,000 cells with single perturbations were subsequently analyzed.

We validated sgRNA calling accuracy by examining target depletion. *Albumin* (*Alb*) sgRNAs caused strong mRNA depletion, likely through nonsense-mediated decay. In Perturb-seq, minimal overlap between *Albumin* expression distributions in targeted versus control cells demonstrated low false positive rates (Figure 3K). We further confirmed fixed-cell Perturb-seq accuracy through a CRISPRi experiment on K562 cells, showing median 87% on-target knockdown (Figure S7A-E). In imaging, cells with *Albumin*-targeting sgRNAs also showed lower *Albumin* mRNA signal than controls (Figures 3O, 3P). Similarly, *Gapdh*-targeting sgRNAs reduced anti-Gapdh immunofluorescence signal (Figures S7F, S7G). Visual inspection suggested that residual *Albumin* mRNA and anti-Gapdh signals likely arose from neighboring non-targeted cells due to imperfect 3D cell segmentation. Accordingly, perturbation readout by RCA-MERFISH also had a low false-positive rate.

We analyzed phenotypic effects using energy-distance permutation tests with stringent multiple testing correction^66^. In Perturb-seq, 109/406 targeting sgRNAs significantly altered transcriptional states, versus 0/50 control sgRNAs (Figure 3L). In morphological imaging, 84/406 targeting and 3/50 control sgRNAs caused significant effects (Figure S7H). Pairs of sgRNAs targeting the same gene showed highly correlated transcriptional and morphological phenotypes (Figures 3M and 3Q).

Perturbations with largest transcriptional phenotypic impacts included genes essential for viability (e.g., *Atp2a2*, *Aars*, *Sf3b6*) and signaling (e.g., *Vhl*) (Figure 3N). Many of these genes also significantly altered morphological states (Figure 3R; hypergeometric *p* < 10^−13^). Overall, perturbation effects correlated positively between transcriptional and morphological measurements (Figure S7I, Pearson’s *R* = 0.5), despite probing different phenotypes.

Notably, while most sgRNAs affected the expression or morphological state of relatively few genes, some perturbations, like those of essential nuclear genes (*Sf3b6*, *Sbno1*, *Polr1a*, *Kin*), impacted several hundred genes’ expression and many imaging targets (Figure S7J, S7K), reflecting pleiotropic consequences of disrupted transcription and RNA processing.

### Multimodal *in vivo* screening with strong phenotypes

**Figure 4:**
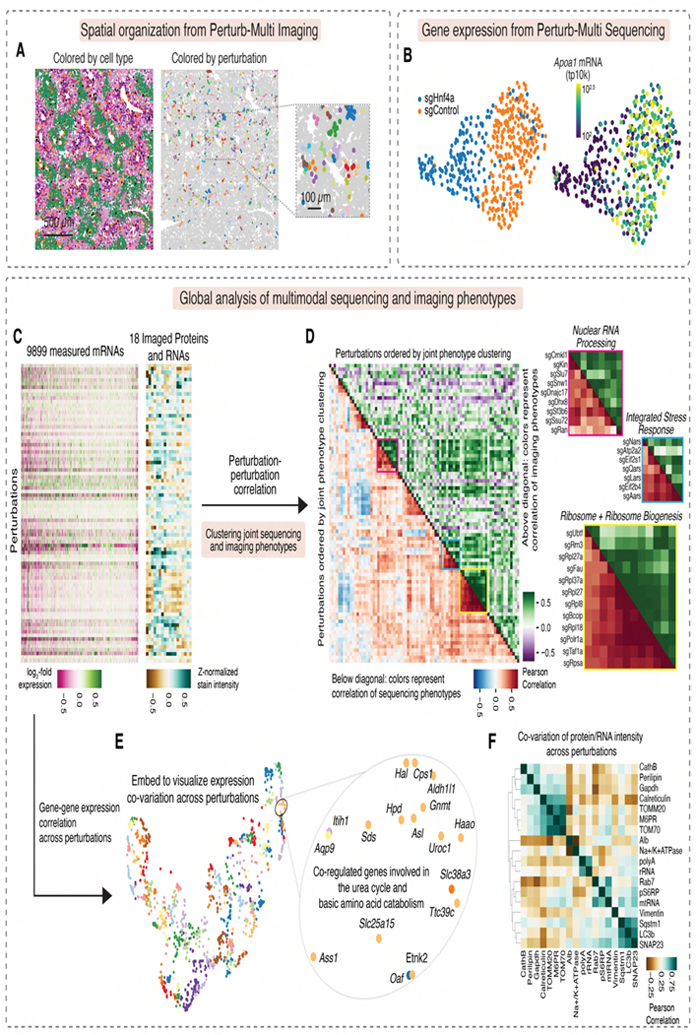
Multimodal *in vivo* screening with strong phenotypes A. Spatial distribution of sgRNAs in the imaging dataset showing proliferation of infected cells. Cells are colored by cell type (left; as in Fig. 2B) or by sgRNA barcode identity (right and zoom). B. A UMAP generated from transcriptome profiles of cells with sgRNAs targeting *Hnf4a* and from a random sub-sampling of cells with control sgRNAs, colored by sgRNA identity (left) or by *Apoa1* expression (right). C. Heat map representation of pseudobulk transcriptional changes (log2-fold change measured by sequencing, left) and staining protein and RNA level changes (Z-normalized changes measured by imaging, right) associated with each sgRNA, relative to cells with control sgRNAs. The colormaps are clipped for visual emphasis. D. Perturbation-perturbation correlation of RNA and protein changes associated with active sgRNAs (left) and zoom-in of the color boxed regions (right). Colors in the heatmap represent Pearson correlation of perturbed gene-level pseudobulk phenotypes measured by sequencing (below diagonal) or imaging (above diagonal). Genetic perturbations are ordered by hierarchical clustering of joint sequencing and imaging phenotype vectors. E. Minimal distortion embedding. Each dot represents an mRNA expressed in hepatocytes. mRNAs that are co-varying in expression across the perturbations are placed in proximity. F. Heat map of the correlation between the expression levels of indicated proteins/RNAs across perturbations, in the imaging dataset. Imaging channels are ordered by hierarchical clustering.

Spatial distributions of perturbed cells showed local colonies of the same perturbation (Figure 4A). Visualization of the Perturb-seq transcriptional phenotypes on UMAP showed strong effects of some perturbations, such as that of the core hepatocyte transcription factor Hnf4a (Figure 4B). Unbiased clustering of our multimodal phenotype vectors identified perturbations causing similar phenotypes across both transcriptional and morphological modalities (Figures 4C, 4D), revealing groups of genes with correlated phenotypes, including clusters of ribosomal proteins and biogenesis genes, nuclear mRNA processing genes, and genes whose disruption activates the integrated stress response. Sequencing and imaging data identified complementary perturbation relationships (Pearson’s *R* = 0.42 for correlation-of-correlations).

We also identified co-varying mRNA or protein phenotypes across perturbations. Using minimum distortion embedding to visualize mRNA expression co-variation (Figure 4E), we identified clusters of co-regulated genes with similar biological functions, including genes involved in the urea cycle and amino-acid metabolism. Similarly, we identified proteins with co-varying intensity, including mitochondrial proteins Tomm20/70, lysosomal protein M6PR, and ER chaperone calreticulin (Figure 4F).

### Identifying candidate genetic regulators of liver physiology

**Figure 5:**
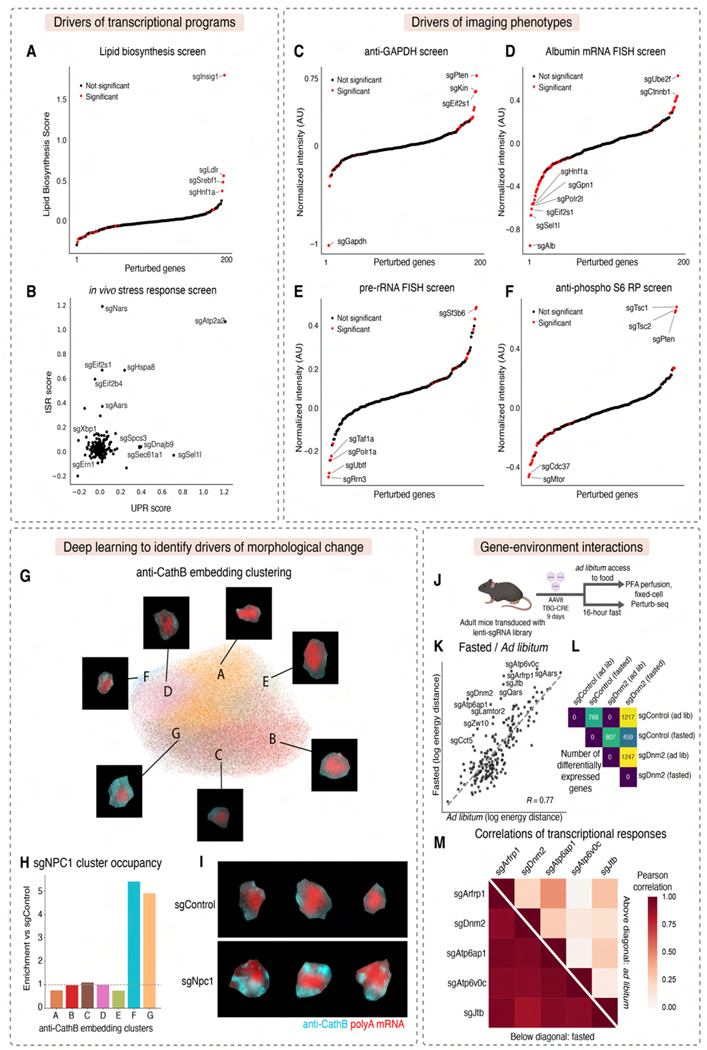
Identifying candidate genetic drivers of liver physiology A. Perturbed genes ranked by their impact on a set of lipid and cholesterol biosynthesis genes (including *Hmgcs1, Sqle*, and *Fasn*) in the Perturb-seq experiment. The y-axis reflects mean log2-fold change of the score, relative to cells with control sgRNAs. Red reflects significance (Benjamini-Yekutieli corrected p < 0.05). B. Scatterplot showing the impact of perturbed genes on literature-defined sets of UPR genes (e.g., *Hspa5* and *Herpud1*) and ISR genes (e.g., *Atf4* and *Ddit4*). The x- and y-axes reflect mean log2-fold change of the scores, relative to cells with control sgRNAs. C. Perturbed genes ranked by their impact on anti-GAPDH intensity in the imaging experiment. The y-axis represents z-scored change relative to cells with non-targeting control sgRNAs (STAR Methods). Red reflects significance (Benjamini-Yekutieli corrected p < 0.05). D. Same as (C) but for *Albumin* mRNA FISH intensity. E. Same as (C) but for pre-rRNA FISH intensity. F. Same as (C) but for anti-Phospho S6 ribosomal protein intensity. G. Leiden-clustered UMAP representation of CathB embeddings from the imaging experiment. Every point represents an individual cell. Example cells from each cluster are shown as insets. H. Bar plot of enrichment in each of the clusters in *Npc1* knockout cells, relative to cells with control sgRNA. I. A sampling of cells with control sgRNAs or *Npc1* sgRNAs showing anti-CathB and polyA FISH signals. J. Schematic of diet + genetic Perturb-seq experiment comparing *ad lib* and fasted conditions. K. Scatterplot comparing the effect of each knockout (measured as energy distance between perturbed cells vs controls) between *ad lib* and fasted conditions. L. Heatmap representing the number of differentially expressed genes between the indicated conditions. differentially expressed genes are defined with Benjamini-Hochberg-corrected, Mann-Whitney p < 0.05, with equal cell numbers per comparison. M. Heatmap representing Pearson correlations of pseudobulk transcriptional responses between the indicated knockouts, in the indicated condition. The knockout-specific transcriptional responses are calculated relative to cells with control sgRNAs from the same mouse. The phenotypes of these knockouts are more correlated in fasted animals (mean Pearson’s *R* = 0.91, vs 0.19 in an *ad libitum* animal).

Using our Perturb-seq data, we identified drivers of key gene expression modules in hepatocytes. For example, we ranked genetic perturbations by their impact on a core program of lipid biosynthesis genes^16^ (Figure 5A) and found that targeting the lipid biosynthesis inhibitor *Insig1* had the strongest effect. *Ldlr* and *Srebf1* knockouts also showed strong positive impacts, possibly through compensatory expression.

We also identified perturbations activating stress response pathways (Figure 5B). The integrated stress response (ISR)^67^ and UPR^68^ were activated by distinct knockouts (Figure 5B), suggesting pathway-specific responses occur *in vivo*, as observed in cell culture^12,16,69^. For example, knockouts of aminoacyl-tRNA synthetase genes (*Nars*, *Aars*) and translation initiation factors (Eif2α/*Eif2s1*, Eif2b/*Eif2b4*) activated ISR. Knockouts of ER import and quality-control genes (*Sec61a1*, *Dnajb9*, *Sel1l*) activated UPR, while knockouts of *Xbp1* and Ire1/*Ern1* decreased UPR gene expression, suggesting a basal level of UPR activation in *ad libitum* mice. Notably, targeting *Atp2a2*, an ER calcium ATPase, strongly activated both UPR and ISR.

We also ranked perturbations by their impact on imaging target intensity. As expected, *Gapdh* and *Albumin* sgRNAs most strongly reduced their respective targets (Figure 5C, 5D). Targeting RNA polymerase II components (*Polr2l*, *Gpn1*) and *Hnf1a*, a transcription factor which binds the *Albumin* promoter, also reduced *Albumin* mRNA FISH, as did targeting the ER-associated degradation (ERAD) factor *Sel1l*. The short duration between Cas9 induction and tissue fixation allowed us to capture acute impacts of perturbations of essential genes. For example, knockouts of RNA polymerase I machinery (*Polr1a, Taf1a, Rrn3, Ubtf*) reduced pre-rRNA intensity (Figure 5E). Unexpectedly, knockout of the nuclear speckle component *Sf3b6* increased pre-rRNA intensity, suggesting interplay between nuclear speckles and nucleoli.

We also identified regulators of signaling pathways important for liver physiology. Knockouts of *Mtor* and *Cdc37*, a co-chaperone potentially involved in mTOR complex biogenesis, decreased pS6RP intensity (Figure 5F), while knockouts of mTOR inhibitors *Tsc1* and *Tsc2* and the growth inhibitor *Pten* increased pS6RP intensity^62,70^, demonstrating the power of imaging for identifying regulators of protein modifications not detectable through sequencing.

Moreover, imaging can uniquely detect complex morphological changes. We performed unsupervised clustering for individual protein channels using their single-cell feature embeddings, illustrated with lysosomal protein CathB as an example (Figure 5G). We identified perturbations that shifted cells between morphological clusters. Knockout of the lysosomal cholesterol transport gene *Npc1* shifted cells to clusters F and G, which show strong anti-CathB punctae (Figures 5H and 5I) and increased anti-CathB intensity (FDR-corrected p < 10^−34^). These results align with the accumulation of dysfunctional lysosomes when lysosomal cholesterol export is blocked—a hallmark of Niemann–Pick disease type C caused by *NPC1*/*NPC*2 mutations in humans^71^.

### Impact of perturbations in altered physiological states

A key advantage of *in* vivo experiments is the ability to identify perturbations sensitive to changes in organismal physiology. A comparison of gene knockout effects in fasted versus *ad libitum*-fed mice using Perturb-seq (Figure 5J) revealed that, while most knockouts produced similar transcriptional changes (Figure 5K, Pearson’s *R* = 0.77), several genes showed strongly enhanced phenotypes during fasting. Specifically, knockouts of autophagy/lysosome genes (*Atp6v0c*, *Atp6ap1*, *Lamtor2*) and endomembrane system genes (*Dnm2*, *Arfrp1*, *Jtb*, *Zw10*) produced stronger differential expression patterns in fasted animals (Figure 5K, 5L), reflecting increased dependence on lysosomal and autophagy function^72^.

Notably, these lysosome/endomembrane knockouts produced more correlated transcriptional responses in fasted mice (mean Pearson’s *R* = 0.91) than in fed mice (*R* = 0.19), suggesting that convergent phenotypes emerge under specific physiological conditions and that diet and nutritional status can rewire *in vivo* genotype-phenotype relationships.

### Three case studies exploring liver physiology with Perturb-Multi

#### Hepatocyte Zonation

Zonated gene expression in hepatocytes contributes to spatial division of liver function^58^. While zonation is believed to be established by gradients of morphogens, oxygen, nutrients, and hormones, the requirements for maintaining zonal gene expression in adult animals remain unclear. We explored mechanisms underlying hepatocyte zonal identity and dynamics.

**Figure 6:**
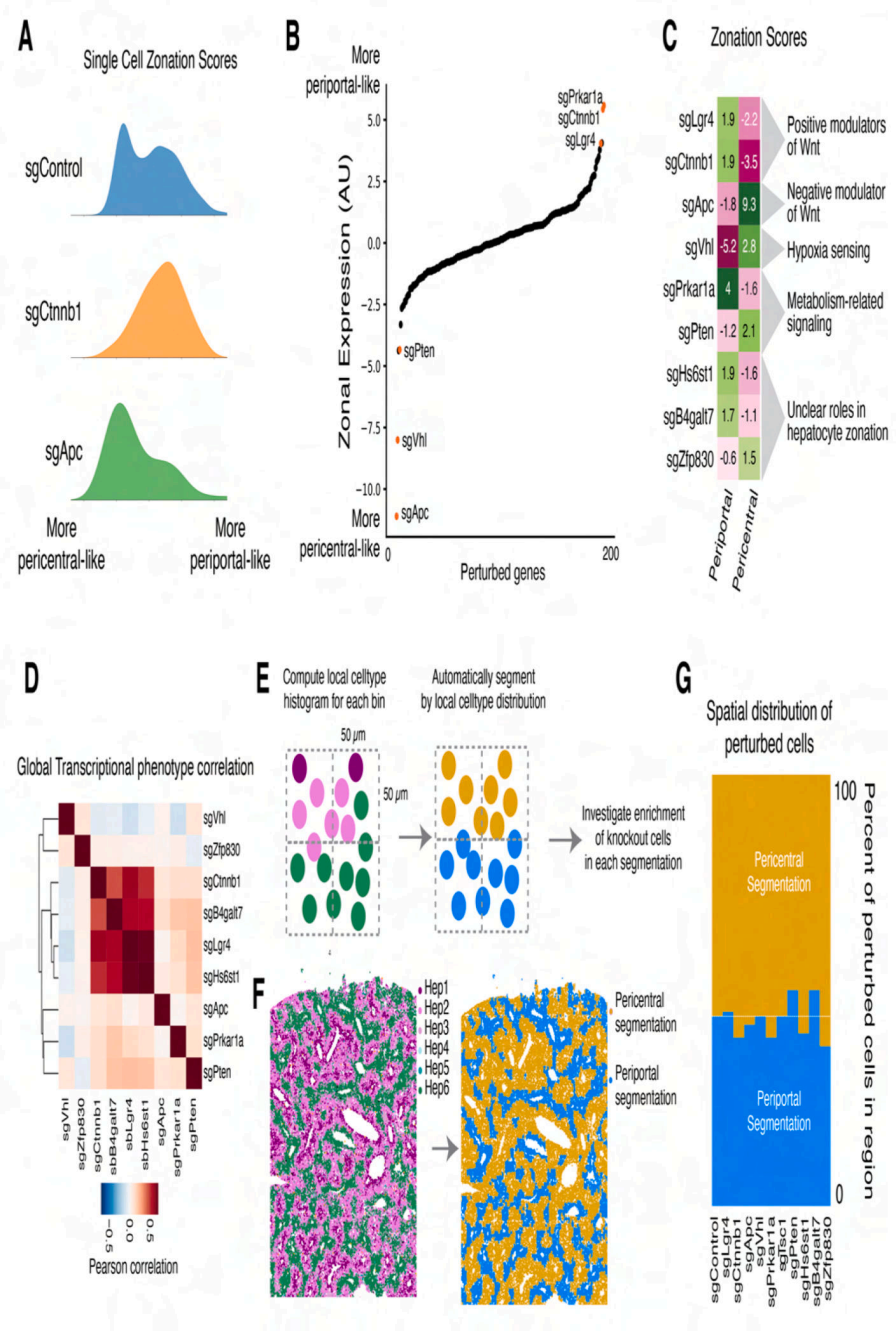
Multimodal investigation of the regulation of hepatocyte zonation A. Kernel density estimate plots showing the distribution of zonation gene expression in cells with control sgRNAs, sgRNAs targeting *Ctnnb1*, and sgRNAs targeting *APC*. The single-cell zonation scores reflect the expression of periportal genes like *Cyp2f2* and *Hal* and pericentral genes like *Glul* and *Cyp2e1*. Periportal and pericentral genes contribute positively and negatively to zonation score, respectively. B. Ranking of perturbed genes by their average impact on zonal gene expression score. C. Heatmap summarizing categories of genes whose perturbation has a large impact on zonated gene expression. Here, the periportal and pericentral expression scores are shown separately. D. Perturbation-perturbation correlation heatmap showing Pearson coefficients of pseudobulk transcriptional changes between indicated sgRNA perturbations. E. Schematic of data-driven zonal segmentation. The proportion of each hepatocyte subtype is calculated in 50-μm x 50-μm bins. The bins are then grouped into two zones based on the local cell-type distribution (STAR Methods) and the enrichment of cells with each perturbation in the two zones is quantified. F. Cell types from RCA-MERFISH (left) and resulting periportal/pericentral zonal segmentation (right). G. Barplot of the fraction of cells in periportal and pericentral zones (as defined above), for the indicated perturbations. The white line represents the fraction of cells with control sgRNAs.

Wnt signaling is an established driver of zonation, with Wnt ligand secretion from central vein endothelial cells promoting pericentral gene expression^58,73^. We scored cells in our sequencing data by zonated gene expression and examined knockouts of Wnt effectors and inhibitors. As expected, knockout of Wnt transducer *Ctnnb1* (β-catenin) decreased the proportion of cells with pericentral gene expression^74,75^, while knockout of Wnt inhibitor *Apc* reduced the proportion of cells with periportal expression^76^ (Figure 6A).

We next examined the effects of all perturbations on periportal and pericentral expression scores (Figure 6B, 6C). Knockout of *Vhl*, which mediates degradation of hypoxia-inducible factors in normoxic conditions, decreased periportal expression and increased pericentral expression, supporting a role for oxygen tension in zonal regulation^58,77,78^. Knockout of *Pten* and *Zfp830*, the latter of which has not been studied in the context of zonation, also shifted cells toward pericentral gene expression. Conversely, *Lgr4* knockout increased periportal gene expression, phenocopying *Ctnnb1*. *Prkar1a* knockout similarly increased periportal expression. Knockout of heparan sulfate synthesis gene *Hs6st1* and proteoglycan synthesis enzyme *B4galt7* also increased periportal expression, possibly by impacting the extracellular matrix in a manner that influences the interactions of Wnt or signaling molecules with their receptors^79,80^.

Comparing transcriptome-wide phenotypes (Figure 6D), we found that targeting of some pro-pericentral factors (*Hs6st1*, *B4galt7*) closely phenocopied *Lgr4* and *Ctnnb1* knockout, suggesting intertwined functions with Wnt signaling. However, targeting *Prkar1a* caused distinct transcriptional responses despite convergent impacts on zonal expression. Knockouts of pro-periportal factors *Apc*, *Vhl*, *Pten*, and *Zfp830* all produced distinct transcriptional phenotypes despite similar impacts on zonal expression, highlighting the complex relationship between cell state and metabolism in hepatocytes.

With sequencing, we measure the endpoint distribution of gene expression, but cannot assess whether cells migrated in response to perturbation. We analyzed our imaging data to determine whether observed expression shifts resulted from trans-differentiation, cell migration, or zone-specific cell death/proliferation. We segmented tissue into pericentral and periportal zones (Figures 6E, 6F) and found that the fraction of cells located in these zones remained unchanged despite gene expression changes (Figure 6G), suggesting that, on the time scale of our experiments, cells underwent trans-differentiation without migration or changes in cell survival or proliferation.

#### Stress Response

**Figure 7:**
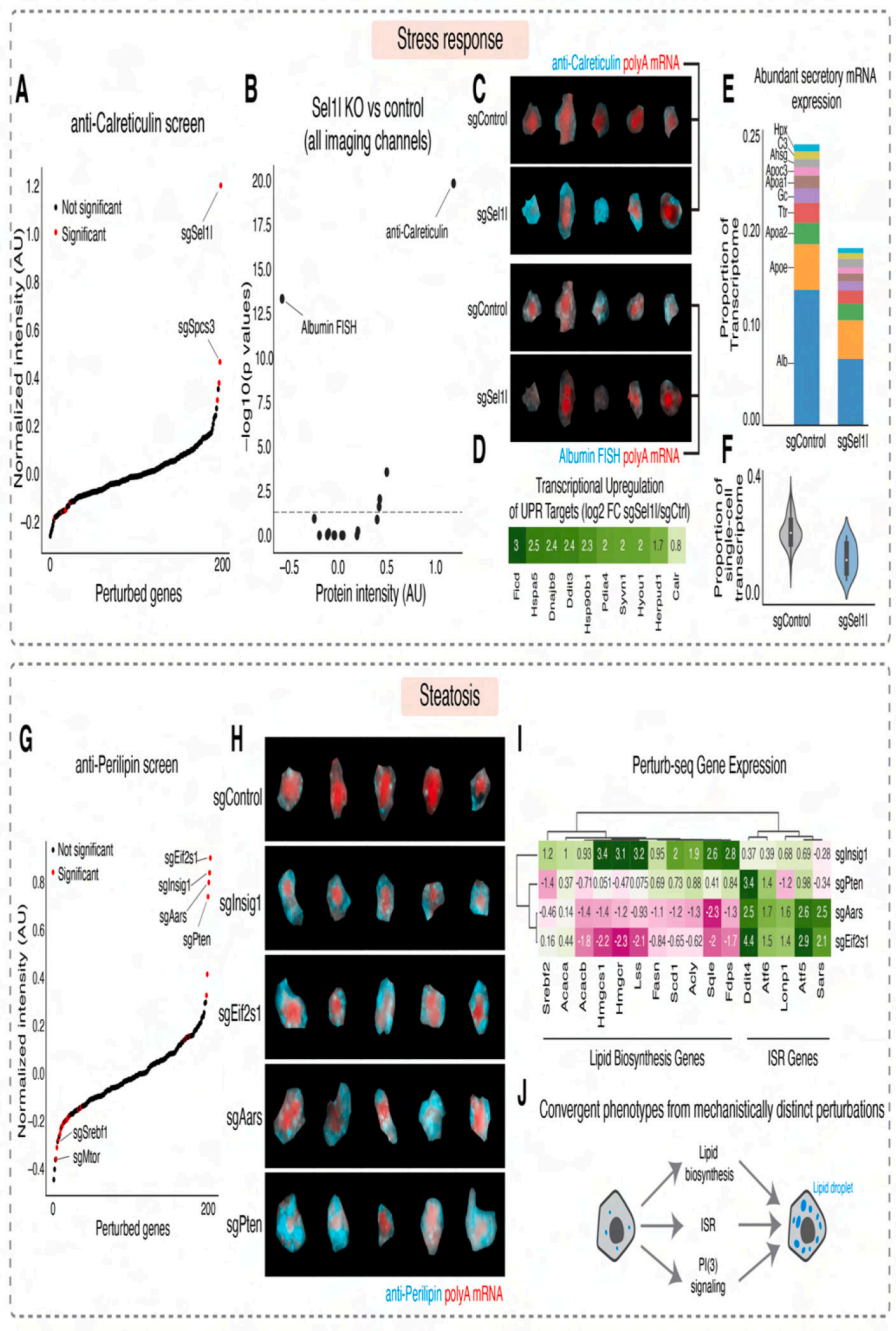
Multimodal investigation of stress response and steatosis A. Perturbed genes ranked by impact on anti-Calreticulin intensity, in the imaging experiment. The y-axis represents z-scored intensity change relative to cells with control sgRNAs (STAR Methods). Red reflects significance (Benjamini-Yekutieli corrected p < 0.05). B. Volcano plot showing intensity change and significance of the imaged protein and RNA channels between cells with sgRNA targeting *Sel1l* vs cells with control sgRNAs. Dashed line indicates corrected p = 0.05. C. An unbiased sampling of cells with control sgRNAs and *Sel1l* sgRNAs, showing the anti-calreticulin channel (top) and the *Albumin* mRNA FISH channel (bottom) alongside polyA FISH channel. D. Heatmaps showing log2-fold change in expression of UPR genes, in the sequencing experiment. E. Stacked bar plot representing the proportions of the ten most abundant secretory mRNAs in the pseudobulk transcriptome. F. Violin plot showing the fraction of each single-cell transcriptome that belongs to mRNAs encoding the ten abundant secretory mRNA, from cells with sgRNAs targeting *Sel1l* and control sgRNAs. G. Perturbed genes ranked by their impact on anti-Perilipin intensity, in the imaging experiment. The y-axis represents z-scored intensity change relative to cells with control sgRNAs. Red reflects significance (Benjamini-Yekutieli corrected p < 0.05). H. An unbiased sampling of cells with control sgRNAs and sgRNAs targeting the indicated gene, showing the anti-perilipin channel alongside polyA FISH. I. Hierarchically-ordered heatmap showing log2-fold change in the expression of lipid biosynthesis genes and ISR genes, for the indicated genetic perturbations. J. Diagram illustrating three convergent mechanisms that cause lipid droplet accumulation: (1) through activation of lipid biosynthesis in the case of *Insig1* knockout; (2) through sequestration of free lipids into lipid droplets alongside ISR activation, in the case of *Eif2s1* and *Aars* knockout; and (3) a distinct, *Pten*-associated mechanism that may include uptake in plasma, lipid synthesis increase, and/or sequestration of free lipids.

Hepatocytes secrete abundant plasma proteins. Thus, integrity of the endoplasmic reticulum (ER) is critical to hepatocyte function. Knockout of *Sel1l*, an ERAD co-factor essential for protein quality control at ER^81^, significantly increased Calreticulin protein levels (Figures 7A, 7C), confirming the transcriptional upregulation of UPR targets observed in sequencing data (Figure 5B). Notably, loss of *Sel1l* also decreased *Albumin* mRNA FISH signal (Figures 7B, 7C).

Leveraging our paired imaging and sequencing data, we examined genome-wide transcriptional changes from *Sel1l* knockout. As expected for an ER quality control factor, *Sel1l* knockout upregulated the expression of numerous ER chaperones, including *Hspa5*, *Dnajb9*, and *Calreticulin* (Figure 7D). Notably, *Sel1l* knockout generally downregulated mRNAs encoding abundant secreted plasma proteins (Figure 7E), causing a ~40% reduction in the mRNAs of abundant secreted proteins (Figure 7F). These findings suggest that a key function of the UPR in hepatocytes is to decrease secreted protein mRNAs and hence the burden of their nascent proteins on ER translocon and folding machinery. This reduction could occur through transcriptional downregulation^82^ or post-transcriptional stress responses like Regulated Ire1a-Dependent Decay (RIDD), where the ER stress sensor Ire1 directly cleaves secreted protein mRNAs on the ER surface^83,84^.

#### Lipid Accumulation

Hepatic steatosis, characterized by accumulation of lipids within hepatocytes, is linked to metabolic syndrome and can progress to serious diseases like metabolic dysfunction-associated steatohepatitis (MASH) and fibrosis^85^. Using our imaging data to identify *in vivo* drivers of steatosis, we observed that knockout of *Insig1*, the growth inhibitor *Pten*^86^, the ISR inhibitor Eif2α (*Eif2s1*), and the alanine-tRNA synthetase *Aars* all caused steatosis, as measured by enlargement and signal increase of lipid droplets (Figures 7G, 7H).

Examining corresponding sequencing data revealed remarkably distinct transcriptional responses for each perturbation (Figure 7I). *Insig1* knockout upregulated cholesterol biosynthesis genes (*Hmgcr*, *Fdps*) and fatty acid biosynthesis genes (*Acaca*, *Fasn*), consistent with its function as a lipid biosynthesis inhibitor^87^. Conversely, *Aars* and *Eif2s1* knockouts upregulated ISR target genes (*Ddit4*, *Atf5*) and decreased most lipid biosynthesis genes. *Pten* knockout produced a third, distinct transcriptional response.

We propose that these genetic perturbations cause the convergent physiological phenotype of steatosis through three separate mechanisms (Figure 7J): (1) transcriptional activation of lipid biosynthesis genes (*Insig1* knockout); (2) sequestration of free lipids into droplets (*Eif2s1*/*Aars* knockout), possibly as a component of the ISR to protect stressed cells from disruptive lipid molecules^88–90^; and (3) a distinct *Pten*-associated mechanism potentially involving blood uptake and increased synthesis, sequestration, and/or repurposing of organellar stores^91–94^.

## DISCUSSION

Massively parallel *in vivo* genetic screens with rich phenotypic readouts could provide unique opportunities to dissect regulators of cellular and tissue physiology in their native context^37^. Here, we establish Perturb-Multi, a multimodal, pooled genetic screening approach combining scRNA-seq and highly multiplex protein and RNA imaging-for multimodal phenotyping. Each component of Perturb-Multi—the imaging assay and fixed-cell Perturb-seq—individually represents an advancement in the state-of-the-art. When combined, these complementary assays further unlock insights unattainable through either method alone. We applied Perturb-Multi to create a multimodal, transcriptional and morphological tissue atlas under normal, fasting, and high-fat diet conditions, and a joint imaging and sequencing genotype-phenotype map in the mouse liver.

Perturb-Multi unlocks access to phenomena uniquely observable in intact physiological contexts. With a single experiment, we probed three core aspects of liver physiology—hepatocyte zonation, dynamic stress responses, and lipid droplet accumulation.

### Zonation:

A key question in mammalian biology is the extent to which cell identities in adult tissues are hardwired or dynamically maintained. We used Perturb-Multi to probe regulation of zonal gene expression *in vivo*. Beyond confirming known roles of oxygen-sensing and Wnt signaling in hepatocyte zonation^58^, we identified previously unknown regulators like *Hs6st1* and *B4galt7*, whose impact on zonation is comparable to Wnt pathway perturbations, highlighting the power of systematic exploration. Notably, these factors are involved in the modification of extracellular matrix proteins, demonstrating the capability of *in vivo* screens to explore causal relationships between the extracellular microenvironment and intercellular signaling. By examining the spatial positions of perturbed cells, we showed that changes in zonal gene expression occurred without cell relocation, suggesting that the zonal identity of hepatocytes is not hardwired, but dynamically maintained by constant signaling. These insights could only be obtained by our multimodal measurements of both single-cell transcriptomes and spatial position of cells in tissues. More generally, our results demonstrate the power of Perturb-Multi to dissect the role of signaling pathways in the establishment and maintenance of spatially organized cell identities.

### Stress response:

Another important question is how conserved stress pathways adapt for tissue-specific function. Hepatocytes produce massive amounts of secreted proteins, so UPR mechanisms are critical for liver function. Notably, the UPR is activated in several liver diseases, including viral hepatitis, alcohol-associated liver disease, and MASH^95,96^. We found that knockout of *Sel1l*, a ubiquitin ligase adaptor protein involved in ER-associated degradation, caused upregulation of UPR genes and downregulation of abundant secreted proteins. We thus hypothesize that a liver-specific adaptation of the UPR is to downregulate secretory protein mRNAs in hepatocytes, thereby reducing the burden of their protein products on the ER folding machinery. This may occur through either transcriptional regulation or post-transcriptional mechanisms like RIDD^84^. This phenomenon may partly explain hypoproteinemia in chronic liver disease and illustrates how a conserved pathway can be repurposed for tissue-specific roles.

### Steatosis:

Lipid droplets play a crucial role in hepatocyte energy storage and lipid metabolism. Accumulation of lipid droplets is key to the pathology of metabolic dysfunction-associated steatotic liver disease (MASLD). We found that knockout of *Insig1*, *Eif2s1*/*Aars* and *Pten* caused a convergent steatotic phenotype— the dramatic accumulation of lipid droplets (as revealed by imaging), but entirely distinct transcriptional responses (as revealed by sequencing), suggesting that each knockout induces lipid accumulation through a different mechanism. Our data thus add a new complexity to the understanding of steatosis—the underlying cell states and molecular mechanisms of apparently similar steatotic hepatocytes can be very distinct, spanning lipid biosynthesis regulation, cellular stress response, and growth signaling, and demonstrating the unique power of our multimodal, paired imaging-and-sequencing approach.

These insights suggest that applying Perturb-Multi to other organs could similarly illuminate tissue-specific physiology and pathology. Our sequencing and imaging approaches can be applied to other tissue types and the robustness and adaptability of fixed tissue will greatly lower the barriers to protocol development and widespread adoption. A key consideration is the efficient delivery of genetic perturbations to different tissue types. Lentivirus has been used for perturbation delivery to multiple organs ^25,26,29,30^ and AAV can further expand accessible tissue types^97,98^, albeit with potentially uncertain MOI. Future screens will benefit from advances in perturbation delivery technologies.

Perturb-Multi is highly scalable. MERFISH can measure the RNAs of thousands of genes^35,99,100^, and the number of protein targets can be increased with additional imaging rounds. Perturbations can grow to genome-wide scales. A key requirement for this perturbation scale is the capacity to measure millions of cells, which is feasible for both Perturb-seq and MERFISH. The cost and throughput of Perturb-seq can be managed by superloading droplets^64^ and split-pool approaches^101,102^ (STAR Methods). Whole-organ MERFISH imaging (~10-million cells) has been demonstrated^103^, and advances in microscope design can further increase throughput (STAR Methods).

Large-scale genotype-phenotype maps enabled by Perturb-Multi will drive discovery and power AI models of cellular function, supporting “virtual cell” efforts to model cellular states and transitions^104–106^ and enable robust predictions of perturbation effects^37^. Genome-wide Perturb-seq datasets have already aided predictive modeling ^107^, but currently suffer from the paucity of comprehensive perturbation data and the limited phenotype diversity and cell types measured. Adding rich *in vivo*, multimodal, genotype-phenotype mapping data will bolster these models, enabling physiologically relevant predictions and advancing discovery, cellular engineering, and therapeutic innovations.

#### Limitations of the study

In this work, we perturbed ~200 genes. Future studies with more perturbations will increase the power of comparisons and chances for discovery. We delivered perturbations only to hepatocytes and focused on cell-autonomous effects. Imaging more cells will allow investigations of non-cell-autonomous effects with high statistical power. Increasing target proteins will expand phenotype diversity to cover more cellular structures and signaling pathways. Finally, additional investigations are required to unravel the in-depth mechanisms underlying our findings related to hepatocyte stress response, zonation, and steatosis.

## RESOURCE AVAILABILITY

### Lead Contact:

Further information and requests for resources and reagents should be directed to and will be fulfilled by the lead contact.

### Materials Availability:

Plasmids generated in this study have been deposited to Addgene.

### Data and code availability:

Sequencing data have been deposited with GEO (GSE275483). Cell images are available at Hugging Face: https://huggingface.co/datasets/xingjiepan/PerturbMulti/tree/main. Our pipeline for RCA-MERFISH processing and software for perturbation analysis are available on GitHub: https://github.com/weallen/InVivoMultimodalPerturbation.

## STAR Methods

### EXPERIMENTAL MODEL AND STUDY PARTICIPANT DETAILS

#### Animals

Male and female wildtype and B6;129-Gt(ROSA)26Sortm1(CAG-cas9*,-EGFP)Fezh/J in a C57BL/6J background were used in this study. Mice were obtained from Jackson labs or bred at Harvard or MIT. Mice were maintained on a 12 hr light/ 12 hr dark cycle (14:200 to 02:00 dark period) at a temperature of 22 ± 1oC, a humidity of 30–70%, with *ad libitum* access to food and water unless otherwise noted. Mice were fed *ad libitum* with a diet containing 60% kcal from fat (Research Diets, Inc. D12492i) or standard chow. Animal care and experiments were carried out in accordance with NIH guidelines and were approved by the Harvard University and Whitehead Institute Institutional Animal Care and Use Committees (IACUC). Lentiviral injections were conducted at P1 and AAV-Cre was introduced at ~P30, with analysis 10 days later.

In total, two wildtype animals were analyzed for the *ad lib* experiments; two wildtype animals were analyzed for the overnight fasting experiments; two wildtype animals were analyzed for the high-fat diet experiments; 3 Cas9 animals were analyzed for the *ad lib* genetic perturbation experiments; and 1 Cas9 animal was analyzed for the overnight fasting + genetic perturbation experiment. In each case, 10 um sections were cut for imaging experiments and 100 um sections were cut from the same tissue blocks for sequencing experiments. Within each condition, the samples were computationally pooled for all analyses.

#### Cell lines

HEK 293T/17 (ATCC CRL-11268) cells were cultured in DMEM supplemented with glutamax, HEPES, 10% fetal bovine serum, 100 units/mL penicillin, and 100 mg/mL streptomycin (ThermoFisher Scientific). K562-CRISPRi cells (Gilbert et al, 2014) were cultured in RPMI supplemented with glutamine, HEPES, 10% fetal bovine serum, 100 units/mL penicillin, and 100 mg/mL streptomycin (ThermoFisher Scientific). AML12 cells (ATCC CRL-22540 were cultured in DMEM/F12 medium supplemented with 10% fetal bovine serum, 10 mg/mL insulin, 5.5 mg/mL transferrin, 5 ng/mL selenium, and 40 ng/mL dexamethasone (ThermoFisher Scientific).

### METHOD DETAILS

#### Lentiviral Construct Cloning

The parental mosaic screening vector (pVV1) was generated from a modified CROP-seq vector (pBA950; addgene 122239 ^108^) and from a lentiviral vector used for CRISPR screens in the liver (pLentiCRISPRv2-Stuffer-HepmTurquoise2; addgene 192826 ^25^) using standard methods. First, the EF1a-BFP was replaced by mTurquoise driven by a hepatocyte-specific promoter. Then, the parental mU6 promoter was replaced by an mU6 promoter that did not contain a loxP site ^109^, yielding pVV1.

#### CRISPR Guide Library Cloning

The parental pVV1 vector was digested with BstXI and BamHI and purified by gel electrophoresis. Library elements containing both sgRNAs and their associated barcodes were ordered as eblocks and pooled before cloning. The library elements were synthesized with three different sgRNA constant regions, which decreases recombination between the sgRNA and the barcode during lentiviral packaging ^12^. The library elements were also digested with BstXI and BamHI and purified by gel electrophoresis. The digested library was ligated into digested pVV1. The ligation was purified with a silica column (Zymo) and electroporated into MegaX DH10B T1 R electrocompetent cells (ThermoFisher Scientific) according to the manufacturer’s protocol. The cells were recovered and then directly introduced into liquid culture and maxiprepped the next day. Samples were plated and used to confirm >1000x library coverage during cloning. Colonies were sequenced to confirm cloning fidelity.

#### Lentiviral Preparation

The lentivirus was generated according to standard methods by the transfection of 293T/17 cells with Fugene HD (Promega), our library vector, psPAX2, and pMD2.G. We used ViralBoost (Alstem) according to the manufacturer’s instructions. We harvested supernatant, filtered it through a 0.45-μm PES membrane, and conducted a 10x concentration with PEG and NaCl (Lenti-X Concentrator; Takara) according to the manufacturer’s instructions. We then pelleted this concentrate by centrifugation in a swinging bucket centrifuge (25,000*g*, 2 hours, 4˚, Beckman Coulter SW 32 Ti) and resuspended it in cold PBS + 4% glucose, leading to an additional 100x concentration. Lentivirus was flash frozen and titered approximately through the transduction of AML12 cells.

#### Mosaic Liver Preparation

The lentivirus was thawed on ice and up to 50ul was injected into the temporal vein of postnatal day one mice ^25,65^. We injected approximately 1 × 10^7^ titer units of lentivirus per animal. We then allowed the mice to grow to adulthood (>P30) and induced Cas9 through the retro-orbital injection of AAV8 with Cre driven by a hepatocyte promoter (Addgene 107787-AAV8; ~5 × 10^11^ genome copies per animal). We maintained the mice for ten days, or alternatively maintained for nine days and then fasted for 16 hours. The mice were anesthetized and fixed by perfusion of PBS + 4% paraformaldehyde. Livers were removed and fixation was continued for 3 hours in PBS + 4% paraformaldehyde, followed by ~13 hours in PBS + 4% paraformaldehyde + 30% sucrose. Fixed livers were then frozen in Optimal Cutting Temperature compound (OCT) and stored at −80˚.

#### Mosaic Liver Dissociation for Fixed Cell scRNA-seq

100μm sections of liver were generated on a cryostat and stored at −80˚ for later processing. The sections were washed with cold 0.5x PBS to remove residual OCT and then resuspended in warm RPMI + 1 mg/ml Liberase Th. The material was transferred to a gentleMACS C tube and dissociated on a gentleMACS Octo Dissociator with heaters (Miltenyi) with the program 37C_FFPE_1. The cells were strained through a 30μm pre-separation filters (Miltenyi) and singlets (diet experiment) or GFP+, mTurqiouse+ singlets (Perturb-seq experiment) were isolated by FACS (ARIA II, BD) and maintained at 4˚ in 0.5x PBS until scRNA-seq.

#### sgRNA probes for Fixed Cell Perturb-seq

Probes for the sgRNAs were obtained from IDT as opools. Probe sequences are found in Table S6. The left-hand side probes targeted the sgRNA constant region; three variants of this probe were included in each hybridization, targeting each of the three constant regions included in the sgRNA library. The left-hand side probes also had a sample barcode sequence. The right hand side probes were 5’ phosphorylated and targeted the protospacer sequence directly. The barcode spike in probes contained the appropriate TruSeq sequences such that they could be amplified separately from the transcriptome probes and sequenced independently. We included a variable number of N bases to ensure base diversity during sequencing.

#### Fixed Cell scRNA-seq Library Preparation and Sequencing

scRNA-seq was conducted with the Single Cell Gene Expression Flex platform (10x Genomics). Spike-in probes for the sgRNAs were included to a final concentration of 2 nM. Cells were counted on a Countess II (ThermoFisher Scientific). We used four sample barcodes and recovered the cells across one (diet experiment) or eight (Perturb-seq experiment) of microfluidic channels. mRNA libraries were prepared according to the manufacturer’s instructions. sgRNA libraries were prepared with the Fixed RNA Feature Barcode Kit according to the manufacturer’s instructions. Libraries were sequenced using a NovaSeq 6000 (Illumina).

#### scRNA-seq Alignment and Calling

The scRNA-seq mRNA data was aligned with CellRanger (10x Genomics). The sgRNA reads were aligned with a custom pipeline using the cell barcodes produced by CellRanger. Briefly, we used bowtie2 (flags --very-sensitive --local) to align the reads to the sgRNA probe library. We then selected sgRNA reads with a cell barcode that was shared with a cellranger-called cell and identified the number of UMIs for each sgRNA in each cell. We tested several perturbation calling approaches including mixed model calling and identifying outliers in a Poisson distribution or zero-inflated Poisson distribution and found the best performance across sgRNAs with thresholding, choosing thresholds empirically to maximize on target knockdown of perturbation targets with apparent NMD. We identified all cells with exactly one sgRNA over the chosen threshold and excluded the rest from all analyses.

#### Antibody Labeling

Antibodies were obtained in >50 ug quantities and labeled with bifunctional 5’ Acrydite - bit sequence - 3’ DBCO oligos (IDT) by enzymatic modification and click chemistry (SiteClick Antibody Azido Modification Kit, ThermoFisher) according to the manufacturer’s instructions. Antibody-oligo conjugates were concentrated in PBS by ultrafiltration with a 100kDa membrane (Millipore), which also removed residual un-conjugated oligonucleotides. Antibody-oligo conjugates were aliquoted into tube strips and snap frozen in liquid nitrogen.

#### RCA-MERFISH Readout Probe Synthesis

Amine-modified 15mer oligonucleotides were obtained from IDT (standard desalting). Oligos were resuspended to 300 μm in 112 mM sodium bicarbonate solution (ThermoFisher). 300 mM Sulfo-Cy3-NHS ester, Sulfo-Cy5-NHS ester, and Sulfo-Cy7-NHS ester (Lumiprobe) solutions were made in dry DMSO (Sigma Aldrich). The appropriate dye was added to each oligo to a final concentration of 10 mM and the dyes were allowed to react for 24 hours in the dark at room temperature. Sodium acetate pH 5.5 was added to a final concentration of 500 mM and then ice cold ethanol was added to a final concentration of 80%. The oligos were incubated at −20˚ C for >24 hours, pelleted by centrifugation at >18,000*g* at 4˚ C for >20 minutes, washed 3x with ice cold 80% ethanol, dried in a vacufuge, and resuspended in TE pH 8 to a final concentration of 100 μm. Labeled oligos were stored at 4˚ C until use.

#### RCA-MERFISH Encoding Probe Design and Construct

For the 205 endogenous genes, we used a 21-bit, Hamming Weight 4, Hamming Distance 4 codebook for MERFISH imaging. For the 456 perturbation barcodes, we used an 18-bit, Hamming Weight 6, Hamming Distance 4 codebook for MERFISH imaging. For both the endogenous genes and perturbation barcodes, individual genes/barcodes were randomly assigned to codewords in the codebook. For each gene/barcode, we designed a total of 8 (endogenous genes) or 3 (barcodes) encoding probes targeting the gene or barcode (Table S1) mRNA sequence. Following the guidelines for MERFISH probe design as previously described ^110^, we selected 60 mer regions that could be split into two 30 mers, where each half had GC content between 30 and 70%, melting temperature Tm within 60–80oC, isoform specificity index between 0.7 and 1, gene specificity index between 0.75 and 1, and no homology longer than 15 nt to rRNAs or tRNAs. Pairs of adjacent probes that had a ligation junction with a G or C at the donor (5’ phosphorylated) end of the probe were excluded. The two halves of the probes were then split, and between them was added an RCA primer sequence and the reverse complements of the 4 (endogenous) or 6 (barcode) readout sequences that encoded the identity of that gene. PCR handles with BciVI (left hand side) and BccI (right hand side) restriction sites were then appended to either end of the probe. The readout sequences on the encoding probes are detected with dye-labeled readout probes with complementary sequences in order to decode the gene or barcode.

#### RCA-MERFISH Encoding Probe Synthesis

RCA-MERFISH encoding probe libraries were first synthesized at femtomolar scale in a pool by Twist Biosciences. Probe libraries were then amplified by limited-cycle PCR (Phusion Polymerase, New England Biolabs), purified using SPRI beads (Beckman Coulter), and then blunt-end ligated using T4 DNA to circularize. Circularized DNA molecules were nicked using Nt.BbvCI (NEB), and then RCA amplified overnight at 30oC using Phi29 DNA polymerase from the nick site. RCA amplified DNA was ethanol precipitated and resuspended in CutSmart buffer. Oligos with degenerate ends containing restriction enzyme sites were annealed to the RCA product, and the mixture was digested overnight with BccI and BciVI (New England Biolabs). The final libraries were then purified using magnetic beads, eluting in Tris-EDTA (TE) (ThermoFisher) buffer to a final concentration of ~10 nM/probe. The resulting library was stored at –20oC until use.

#### RCA-MERFISH Sample Preparation

Sample preparation occurred over several days. Briefly, after perfusion fixation and cryopreservation, we cut thin sections of tissue, decrosslinked the tissue, and stained it with a pool of oligo-conjugated antibodies targeting diverse subcellular organelles, membrane, and proteins. After antibody staining, we modified all RNA and DNA in the tissue with MelphaX, and embedded the sample in a thin acrylamide gel, such that cellular RNAs, sgRNAs with barcodes, and antibody-associated DNA oligonucleotides were covalently linked to the gel. We then digested the proteins and washed away lipids to both clear the tissue and make the remaining RNA and DNA accessible to enzymes and probes. After clearing, we hybridized a library of padlock probes targeting both endogenous mRNA and perturbation barcodes. After stringent washing, we then ligated the RNA-hybridized padlock probes and performed RCA. These steps are described in this section. After RCA, the readout sequences on the padlock MERFISH encoding probes were then detected by sequential rounds of hybridization with complementary readout probes as described in the next section.

Blocking, antibody staining, MelphaX modification, probe hybridization, ligation, and RCA were conducted with the coverslips inverted onto small volumes of reaction mixture over parafilm, whereas decrosslinking, washing, and digestion were conducted with the coverslips upright in >5 ml of solution in 60 mm tissue culture dishes. Silanized, PDL-coated coverslips were prepared according to the method of ^47^.

10um sections of liver were cut onto silanized, PDL-coated coverslips, warmed to room temperature for 15 minutes, and attached to the surface by 15 minutes of postfixation in PBS + 4% paraformaldehyde. The sections were washed 3x with PBS and decrosslinked at 60˚ in TE pH 9 (Genemed) for an hour. The sections were then washed with PBS.

The sections were blocked at RT for 20 minutes in blocking buffer (1x PBS, 10 mg/ml BSA (UltraPure, ThermoFisher), 0.3% Triton X-100, 0.5 mg/ml sheared salmon sperm DNA (ThermoFisher), and 0.1 U/μL SUPERase·In RNase Inhibitor (ThermoFisher) ^111^. It was crucial that the blocking buffer did *not* contain dextran sulfate, as even trace amounts inhibited later ligation and/or RCA.

The antibody-oligo conjugates were pooled and diluted into blocking buffer. The sections were then stained overnight at 4˚ with this mix. We estimate that we stained each coverslip with ~150 ng of each oligo-antibody, diluted into 150 ul of blocking buffer. The sections were then washed 3x with PBS and then incubated with PBS at RT for 15 minutes, then postfixed for 5 minutes in PBS + 4% paraformaldehyde. The sections were then washed 3x with PBS and fixed with 1.5 mM BS(PEG)_9_ in PBS for 20 minutes, inactivated in PBS + 100 mM Tris pH 8 for 5 minutes, washed 3x in PBS, and stored in tightly sealed tissue culture dishes at 4˚ in 70% ethanol for 24 hours to one month.

After antibody staining, the samples were then modified with MelphaX ^112^ that was diluted 1:10 in 20 mM MOPS pH7.7 at 37oC for 1 hr. The samples were washed three times with PBS, then embedded in an acrylamide gel (4% v/v 19:1 acrylamide:bis-acrylamide (Bio-Rad), 300 mM NaCl, 60 mM Tris pH 8, 0.2% v/v tetramethylethylenediamine [TEMED], and 0.2% w/v ammonium persulfate [APS]) for 1.5 hrs at room temperature. The samples were then digested at 42oC for 48 hours in digestion buffer (2% v/v sodium dodecyl sulfate [SDS] (Thermo Fisher), 1% v/v proteinase K (New England Biolabs), 50 mM Tris pH 8 (Ambion), 300 mM NaCl (Ambion), 0.25% Triton X-100 (Sigma), 0.5 mM ethylenediaminetetraacetic acid [EDTA] (Ambion)).

After digestion, samples were washed three times with PBS + 0.1% Triton X-100 to remove residual SDS. Samples were then hybridized with a mixture containing 2×SSC, 30% v/v formamide (Ambion), 1% v/v murine RNase inhibitor (New England Biolabs), 0.1% w/v yeast tRNA, 5% w/v PEG35000 (Sigma), 1 uM of a polyA probe, a library of probes for imaging pre-rRNA, mtRNA, and *Albumin* at 1 nM/probe, and each RCA-MERFISH encoding probe library at 1 nM/probe. The polyA probe had a mixture of DNA and LNA nucleotides (/5Acryd/TTGAGTGGATGGAGTGTAATT+TT+TT+TT+TT+TT+TT+TT+TT+TT+T) where T+ is a locked nucleic acid and /5Acryd/ is a 5’ acrydite modification. The samples were then hybridized for 36–48 hours at 37oC in a humidified chamber.

After hybridization, the samples were washed twice for 30 min in 2×SSC, 30% v/v formamide at 47oC. The samples were then washed three times with PBS + 0.1% v/v Tween 20, and once briefly with preligation buffer (50 mM Tris-HCl pH8, 10 mM MgCl2). The RCA-MERFISH probes were ligated with 10% v/v (~1 uM) SplintR ligase (New England Biolabs), 1× SplintR ligase buffer, 1% v/v murine RNAse inhibitor, and 100 nM RCA primer (TCTTCACCCGGGGCAGCTGAA*G*T, where * is a phosphorothioate bond) at 37oC for 1 hr. Samples were then washed three times with PBS + 0.1% Tween 20. Next, samples underwent rolling circle amplification using 1× Phi29 Buffer (Lucigen), 10% v/v Phi29 enzyme (Lucigen), 0.2 mg/ml BSA, 250 μM dNTP (New England Biolabs), 25 μM aminoallyl-dUTP (ThermoFisher), and 1% v/v murine RNase inhibitor for 2 hrs at 37oC. Finally, the samples were washed three times in 1×PBS, and then crosslinked for 30 min with 1×PBS + 1 mM BS(PEG)9 at room temperature, before a final quick wash with 1×PBS. Samples were stored in 1×PBS with 1% v/v murine RNAse inhibitor at 4oC for up to one week.

#### Multiplexed RNA and protein imaging by RCA-MERFISH and sequential hybridization

RCA-MERFISH samples were imaged on a custom epifluorescent microscope with automated fluidics, as previously described for MERFISH imaging^110^. Briefly, samples were mounted in a flow cell (Bioptechs) with a 0.75-mm-thick flow gasket on a Nikon epifluorescence microscope.

For each round of hybridization, mixtures of Cy7-, Cy5-, Cy3-labeled readout probes for each triplet of bits to be read out were diluted to a final concentration of 10 nM/probe in 5 mL of 2×SSC, 10% formamide, 0.1% Triton X-100. The samples were stained for 15 min, then washed with 2×SSC, 10% formamide, 0.1% Triton X-100. Finally, imaging buffer was flowed into the chamber. The imaging buffer consisted of 2×SSC, 10% w/v glucose (Sigma), 60 mM Tris-HCl pH8.0, ~0.5 mg/mL glucose oxidase (Sigma), 0.05 mg/mL catalase (Sigma), 50 μM trolox quinone (generated by UV irradiation of 6-hydroxy-2,5,7,8-tetramethylchroman-2-carboxylic acid (Sigma)), 0.2% v/v murine RNAse inhibitor, and 0.1% v/v of Hoechst 33342 dye (ThermoFisher).

After the readouts were hybridized and imaging buffer added, the samples were imaged with a high-magnification, high-numerical aperture objective. For wildtype animals in Figs 1–2, a 60X 1.4NA oil immersion objective was used, with a pixel size of 108 nm/pixel. For genetically perturbed experiments in Figs 3–7, a 40X 1.3NA oil immersion was used, with a pixel size of 162 nm/pixel. We imaged each field of view (FOV) with a 10-plane z stack with 1.5 μm spacing between adjacent z planes, where each z-plane was imaged in the 750-nm, 650-nm, 560-nm, and 405-nm channels. After each round of imaging, the readout probes were stripped off using 2×SSC, 80% formamide stripping buffer for 10 min, followed by two washes of readout buffer, and one wash of 2×SSC. The different panels (antibodies and structural RNAs, endogenous RNA, and barcode RNA) were imaged back-to-back on the same tissue sections, where protein (labeled with oligo-conjugated antibodies) and structural RNA were first imaged by with sequential rounds of multicolor FISH, followed by endogenous RNAs and barcode RNAs in two separate RCA-MERFISH runs. Each experiment took 36–48 hours depending on the number of fields of view, and whether the perturbation barcode library was imaged in addition to the endogenous RNA and protein panels.

#### Data Processing Pipeline

RCA-MERFISH gene expression was processed using a modified version of the MERlin pipeline, as previously described^110,113^, with the addition of a machine learning filtering step that used XGBoost to train a classifier to discriminate incorrectly decoded molecules that were assigned to blank barcodes and putatively correctly decoded molecules that were assigned to coding barcodes on a subset of the data. This classifier was then applied to the remainder of the data, and only molecules that were classified as coding molecules with an adaptive 5% false-positive threshold were exported for assignment to individual cells. Each library (endogenous RNA or barcodes) were decoded separately.

Cell segmentation was accomplished using a custom Cellpose model that was trained on images of polyA staining for cytoplasm + nucleus, and Na+/K+ ATPase staining for membrane. A separate model was trained for data collected using the 60X and 40X objectives, and applied to the respective datasets. Each cell was assigned a unique identifier and its position, shape, and z extent were recorded. The molecules that were exported from each field of view were then assigned to cells based on overlap between molecules and Cellpose-created mask for each cell in three dimensional space. The total number of molecules of each type for each cell was summed, and the result exported as an AnnData objective.

After decoding and cell assignment, all RCA-MERFISH datasets were concatenated into a single dataset. This was done separately for the wildtype physiological perturbation data (Figures 1 and 2) and the genetically perturbed data (Figures 3–7). Cells were filtered to remove all cells with less than 25 or greater than 1500 molecules per cell, and genes expressed in <3 cells were removed. Each cell’s gene expression values were scaled such that the sum of gene expression values per cell added to 10,000, then was log-transformed. The area and number of molecules per cell were regressed out using linear regression, and the residuals were z-scored. The first 20 principal components were then computed and the data were integrated with log-transformed, z-scored Flex data using Harmony^139^, using the top 40 principal components computed based on the subset of genes measured in RCA-MERFISH. The data were then jointly clustered using Leiden clustering with resolution = 0.4. Clusters with fewer than 10 differentially expressed genes were then greedily merged, and the final set of clusters were manually annotated based on marker gene expression. For perturbation data, the individual guide was called for each cell when there were > 3 molecules per cell for a given barcode.

The labels assigned to clusters in the integrated Flex and RCA-MERFISH data from wildtype animals were transferred to the perturbed data through integration and label transfer. After pre-processing to filter out cells, normalize, log transform, and z-score the data as described above, the two datasets were integrated using Harmony^139^. A K-nearest neighbors classifier was then trained to predict the clusters annotations of the wildtype RCA-MERFISH data from the top 20 principal components of the data, with K=10. This classifier was then applied to the perturbed RCA-MERFISH data to predict cluster identity.

For morphological imaging, each channel used for morphological imaging was adaptively contrast adjusted. A single z-plane in the middle of each segmented cell was selected, and the image of each cell in each channel was cropped out of the larger field of view, in a 300×300 (60X objective datasets) or a 256 × 256 (40X objective datasets) square, using the segmentation for that cell as a mask. The background around each cell outside of the mask within that square was set to zero. The imaging data for each cell was then associated with the unique identifier for that cell, for integration with RCA-MERFISH data for endogenous RNA and perturbation barcodes.

#### Deep autoencoder model

We developed a deep autoencoder model to extract biologically relevant features from single-cell multiplexed protein and abundant RNA images based on the second generation of the vector quantized variational autoencoder (VQ-VAE) ^61^. The model takes advantage of the power of VQ-VAE to learn meaningful representations using self-supervised training. Inspired by the cytoself model ^11^, we used auxiliary classification tasks to guide the model to focus on features of biological importance. Each single-color protein image of a particular protein/RNA channel was concatenated to a fiducial channel to create a two-color image. We used the polyA FISH staining as the fiducial channel to provide information of the relative location of the stained proteins/RNAs to the nuclei. From the two-color image, the model used a ResNet with two residual blocks to generate a bottom level representation as described in the second generation VQ-VAE design. The bottom level representation was then used to generate a top level representation with another ResNet with two residual blocks. Both bottom and top representations were converted to discrete representations by vector quantization^61^. The quantized bottom and top representations were concatenated and decoded by a ResNet with two residual blocks to regenerate the input image. The concatenated representation vectors were fed into a multi-layer perceptron (MLP) classifier with one hidden layer to predict the identity of the protein/RNA channels. The hidden representation of the MLP classifier had 512 dimensions, which was used as the representation vector of the input image. The representation vectors for different protein/RNA channels of one cell were concatenated to generate the morphological representation of a cell. The cell representation was fed into another MLP classifier to predict the transcriptionally defined cell type and/or the diet condition of the cell.

The loss function of the autoencoder model contained 4 terms, namely, the latent loss, the image reconstruction loss, the protein/RNA classification loss and the cell-type or diet-condition classification loss. We used the same latent loss definition as the VQ-VAE model^61^, which measured the difference between the latent representations before and after quantization. The image reconstruction loss was defined as the mean squared error of the reconstructed image compared to the input image. The protein/RNA and cell-type/diet-condition classification losses were defined as the cross entropy loss of classification.

#### Training the deep autoencoder

Images of single-cells were cropped out from the fields of view as square boxes. We set all the pixels outside the target cells to zero using the cell segmentation masks, such that each cropped image only contained one cell. The cropped single-cell images were rescaled to 128×128 pixels and used as input for the autoencoder training. We optimized the autoencoder model with stochastic gradient descent using the Adam optimizer until the loss function converged.

We trained a VQ-VAE model to embed cells under different diet conditions using both the protein classification auxiliary task and the cell classification auxiliary task that predicted transcriptionally defined cell types and diet conditions. For embedding the cells under CRISPR perturbations, because we only included hepatocytes, the autoencoder model was trained using only the protein classification auxiliary task.

#### Tissue zone segmentation

The zonal segmentation of tissue in Figure 6E-G was accomplished by computing for each replicate a 2D histograms at 50 μm resolution of the number of Hep1+Hep2 or Hep5+Hep6 cells in each bin. These histograms were then blurred with a Gaussian filter with sigma = 0.5 and normalized by dividing by the maximum across all bins. For each bin, the zone was determined as whether the normalized Hep1+Hep2 or Hep5+Hep6 count was greater for that bin.

#### Scalability of Perturb-Multi Experiments

In these initial experiments, we performed Perturb-Multi experiments at the scale of hundreds of perturbations, sequenced in tens of thousands of perturbed cells, and imaged hundreds of thousands of cells, including tens of thousands of perturbed cells. In order to perform genome-wide experiments, order-of-magnitude increases in the number of sequenced and imaged cells will be required. Here, we describe some simple extensions of Perturb-Multi that will allow for genome-wide experiments to be performed.

##### Scaling sequencing throughput:

The primary limitation to scaling Perturb-seq is the cost of reagents for single-cell library preparation and sequencing. Using 10x Genomics Flex-based scRNA-seq, substantial gains can be achieved by superloading the number of cells per 10X lane. By pooling cells that have been barcoded with *N* distinct barcode pools, the same number of barcoded droplets can be superloaded by a factor of *N* with approximately the same doublet rate. Using the latest version of 10X Flex technology at the time of publication (GEM-X), it should be possible to load 2.56 million cells using 16 barcodes per 8-lane kit, at a cost of less than $0.01 per cell. This represents a substantial cost reduction compared to previous large-scale Perturb-seq experiments, such as our earlier genome-scale Perturb-seq work in K562 cells^16^.

Split-pool-based methods (e.g., sci-RNA-seq, Split-seq) are considerably more scalable than droplet-based methods, though they currently offer substantially lower RNA sensitivity in published studies. Sequencing costs have decreased by a factor of two in recent years, and we anticipate that widespread adoption of split-pool technologies available from several commercial sources will drive further >2x sequencing cost reductions in the near future.

##### Scaling imaging throughput:

Unlike sequencing, imaging-based experiments are not substantially limited by reagent cost. However, increasing the number of perturbations measured per experiment requires imaging more cells, which in turn requires imaging larger sample areas and thus increases total imaging time.

The total time for an imaging experiment depends on the number of imaging rounds $\left(N_{rounds}\right)$, the constant time per round for fluidic exchanges $\left(T_{fluidics}\right)$, and the number of fields of view imaged $\left(N_{fov}\right)$. For each field of view, the imaging time depends on the number of z-planes $\left(N_{zplanes}\right)$, the number of color channels $\left(N_{colors}\right)$, the exposure time per color per z-plane $\left(T_{exposure}\right)$, and the stage movement time $\left(T_{movement}\right)$ when shifting to a new field of view:

$$N_{rounds}\left(T_{fluidics}+N_{fov}\left(T_{movement}+T_{exposure}\times N_{colors}\times N_{zplanes}\right)\right)$$

Although simply increasing the total imaging time is the most straightforward way to image more cells, reducing imaging time (i.e., increasing throughput) is desirable. The simplest approach is to lower the objective magnification from our current 40X to 25X or 20X, enabling more cells to be imaged per field of view and thereby reducing the total number of fields required. High numerical aperture objectives exist at 1.05 NA (silicone-oil immersion), 1.0 NA (water-immersion), and 0.8 NA (air), all of which can be coupled with these lower magnifications. Using a 25X or 20X objective, combined with cameras that have more pixels (and typically smaller pixel sizes), would increase throughput by approximately 2.56-fold and 4-fold, respectively. In combination with optimizations to stage movement time, time spent on staining and stripping, and reductions in exposure time (for example, with the help of machine learning), we anticipate that throughput improvements by 5–10 fold (or possibly more) are achievable.

Finally, because imaging experiments must measure both perturbed and unperturbed cells within a tissue section, simply increasing the fraction of perturbed cells by increasing the viral multiplicity of infection can significantly enhance the throughput of experiments—even when using the same hardware. This approach would also increase the fraction of cells infected by multiple viruses, thus carrying multiple distinct perturbations. Since our method allows for unambiguous identification of perturbations even in multiply-infected cells, these experiments offer the additional advantage of revealing the combined effects of multiple perturbations, as well as the effects of single perturbations.

### QUANTIFICATION AND STATISTICAL ANALYSIS

#### scRNA-seq Analyses

##### Energy Distance Calculation and Permutation Testing

Energy distances and permutation tests were calculated according to the method of ^66^. We used the first 20 PCs generated from the tp10k-normalized, log1p-transformed data. We used the Holm-Šídák multiple testing correction for our permutation testing. For the fasted vs *ad libitum* comparisons, we compared the transcriptional states of perturbed cells to those of cells with control sgRNAs in the same mouse in the same condition.

##### Z-scoring relative to control

For some purposes, we analyzed z-scored transcriptional data. We calculated the z-scores by tp10k-normalizing the data, identifying the mean and standard deviation for each mRNA in control cells, and then used these values to identify the z-score for each gene in each cell, relative to control cells. We performed this calculation separately for each barcode in each GEM group and then concatenated the cells to form a final z-normalized expression matrix. This transformation emphasizes changes in mRNAs with low variance in control cells and may decrease batch effects between barcodes and GEM groups.

##### Pseudobulk Correlation Calculations

Correlations calculated between pseudobulk transcriptional responses are either Pearson correlations calculated from mean log-transformed transcriptional responses, with the expression in controls subtracted to a Perturbation-associated phenotype, or correlations of mean Z-scored transcriptional changes. We did not observe significant differences between the two. To decrease noise, we only included genes with high expression in control cells (highest 250 or highest 1000) in the calculations. In Figure 3M, we show sgRNAs targeting genes that have two sgRNAs that have substantial transcriptional phenotypes.

##### Differential Expression

We used Benjamini-Hochberg-corrected Mann-Whitney testing to identify genes whose expression is significantly affected by perturbations (corrected p < 0.05). We used tp10k-normalized, log1p-transformed data for these calculations. The heat map showing changes in expression (Figure 7D) represents log2-fold changes of pseudobulk expression versus cells with control perturbations.

##### Scoring and significance testing

The scores in Figures 5A and 5B are calculated using gene sets derived from a previous large-scale perturbation experiment in cell culture ^16^. The scores are calculated as the pseudobulk z-scored change relative to control cells, averaged for all genes in the gene set, for all cells with each perturbation. The scores in Figures 6A, 6B, and 6C are calculated with a literature-curated set of zonation markers and reflect the sum of pseudobulk or single-cell z-scored changes relative to control cells. The periportal markers are *Cyp2f2*, *Hal*, *Hsd17b13*, *Sds*, *Ctsc*, *Aldh1b1*, and *Pck1* and the pericentral markers are *Cyp4a14*, *Cyp2d9*, *Gstm3*, *Cyp4a10*, *Mup17*, *Slc1a2*, *Slc22a1*, *Cyp1a2*, *Aldh1a1*, *Cyp2a5*, *Gulo*, *Cyp2c37*, *Lect2*, *Cyp2e1*, *Oat*, *Glul*. Periportal expression contributes positively to the score and pericentral contributes negatively to the score, or alternatively they are shown separately. Significance is calculated relative to negative control cells by Mann-Whitney with Benjamini-Yekutieli correction for multiple testing (corrected p < 0.05).

#### Image Intensity Analysis

##### Z-scoring relative to control

When analyzing intensity, we z-scored each protein/RNA channel relative to the mean and standard deviation of that channel in control cells (cells with control sgRNAs), from that same imaging sample. We then concatenated the cells from the various imaging samples to form a final z-normalized intensity matrix. This transformation emphasizes changes in protein/RNA intensities with low variance in control cells and may decrease batch effects between imaging samples.

##### Pseudobulk Correlation Calculations

Correlations calculated between pseudobulk protein/RNA intensity responses are Pearson correlations calculated from mean z-scored intensity responses. In Figure 3Q, we only show perturbations targeting genes that have two sgRNAs that are significant in a corrected energy distance permutation test.

##### Number of Protein/RNA Channels Exhibiting Differentially Intense Signals

To quantify the number of differentially intense protein/RNA channels (Figure S7K), we used Benjamini-Hochberg-corrected Mann-Whitney testing (corrected p < 0.05) on the z-scored intensity data.

##### Intensity Scoring and significance testing

The scores in Figures 5C, 5D, 5E, 5F, 7A, 7B, and 7G are calculated as the mean z-scored change relative control cells for all cells with each perturbation. Significance is calculated relative to negative control cells by Mann-Whitney with Benjamini-Yekutieli correction for multiple testing, corrected p < 0.05.

#### Clustering and genotype-phenotype mapping

The perturbation heatmap in Figure 4D is generated from the hierarchical clustering of a joint vector including Pearson correlations of pseudobulk log-transformed transcriptional responses measured by sequencing and pseudobulk z-scored staining intensity changes measured by imaging (Euclidean distance metric, UPGMA algorithm). The representation of gene co-regulation in Figure 4E is generated from correlations between z-scored pseudobulk expression levels of pairs of genes across perturbation (the transpose of the transcriptional component of the data used to generate the correlations in Figure 4D). The position of spots derives from a two-dimensional minimal distortion embedding of these correlations that tries to place co-regulated genes in proximity. The color of spots derives from a density-based clustering of a separate twenty-dimensional minimal distortion embedding of co-expression, calculated according to the method used for mRNAs in Figure 4B of ^16^.

## Supplementary Material

1 Figure S1: Development and optimization of the RCA-MERFISH protocol, related to Figure 1.
A. Phi29 used in RCA degrades ssDNA FISH probes. Left: Single-molecule FISH signal in U-2 OS cells without Phi29 treatment. Center: Single-molecule FISH signal in U-2 OS cells pre-treated with Phi29 at 37oC for 1 hr before FISH staining. Right: Single-molecule FISH signal in U-2 OS cells treated with Phi29 at 37oC for 1 hr after FISH staining
B. Quantification of the effect of Phi29 on ssDNA FISH probes from (A), in spots/cell.
C. Quantification of the effect of Phi29 on ssDNA FISH probes from (A), in intensity/spot.
D. Dextran sulfate (crowding agent) inclusion in hybridization buffer inhibits Phi29 enzymatic activity. Left: RCA-MERFISH signal with padlock probe against GFP in U-2 OS cells not expressing GFP, detected by readout probes complementary to readout sequences on the padlock. Center: RCA-MERFISH signal with padlock probe against GFP in U-2 OS cells expressing GFP, with dextran sulfate in the hybridization buffer. Right: RCA-MERFISH signal with padlock probe against GFP in U-2 OS cells expressing GFP, without dextran sulfate in the hybridization buffer.
E. Optimization of alternative crowding agents to dextran sulfate. Multiple additives to hybridization mixture, staining U-2 OS cells expressing either GFP or mCherry with a single probe against GFP, in terms of number of spots per cell, distinguishing GFP+ (signal) and mCherry+ (background) cells. Peg8k = Poly(ethylene glycol) average mol wt 8,000, Peg35k = Poly(ethylene glycol) average mol wt 35,000, Dextran = unsulfonated dextran; all are added to the hybridization solution so the final w/v is at the indicated percent.
F. RCA amplicons of RCA-MERFISH probe against GFP in U-2 OS cells expressing either GFP or mCherry. These data are from the PEG35K 5% condition. There are many more GFP amplicons in GFP-expressing cells than there are in mCherry-expressing cells, indicating the specificity of RCA-MERFISH. The specificity is quantified in Figure S1E.
G. Optimization of RCA-MERFISH processing order. In one iteration, samples were stained with padlock probes and exposed to ligation and RCA enzymes prior to gel embedding and protease digestion. In the other iteration, RCA amplification occurred after gel embedding and protease digestion. The bar plot shows the number of amplicons per field of view across the first two bits, in Cy7 and Cy5 channels, from a 120-gene RCA-MERFISH library.
H. Optimization of RCA-MERFISH staining protocol. In one iteration, samples were stained with padlock probes before gel embedding and protease digestion. In the other iteration, samples were stained with padlock probes after gel embedding and protease digestion. The bar plot shows the number of amplicons per cell across the first two bits, in Cy7 and Cy5 channels, from a 120-gene RCA-MERFISH library.
I. Optimization of RCA-MERFISH digestion protocol. Samples were digested with detergent and proteinase K at the indicated temperature, with or without an initial decrosslinking step. The bar plot shows the number of amplicons per cell across the first two bits, in Cy7 and Cy5 channels, from a 120-gene RCA-MERFISH library.
J. Optimization of RCA-MERFISH and immunofluorescence across different decrosslinking conditions. The RCA-MERFISH performance is quantified by amplicon counts per cell for a single bit, using an RCA-MERFISH library at low concentration (~0.1 nM/probe), hence the lower counts per cell than (I) which used ~10X higher probe concentration. The immunostaining performance is quantified by measuring the intensity of staining with an anti-Tomm20 oligo-antibody. The conditions tested are (1) no decrosslinking (no deX), (2) decrosslinking at 47˚ for 30 minutes, (3) decrosslinking at 60˚ for 30 minutes, (4) decrosslinking at 70˚ for 30 minutes, and (5) decrosslinking at 85˚ for 15 minutes. After poor initial RNA results with traditional citrate pH 6, all decrosslinking was conducted in TE pH 9.
K. Fluorescence micrographs of RCA-MERFISH and immunofluorescence performance, without decrosslinking and after decrosslinking optimization.
L. Individual and cumulative increase in RCA-MERFISH performance achieved by each optimization, including hybridization buffer / crowding agent optimization, post-gel RCA, post-gel probe staining, in-gel digestion optimization, decrosslinking (Dex) optimization.
M. The number of decoded RCA-MERFISH mRNA counts per hepatocyte in the final, optimized datasets, measured with our 209-gene library.
N. Correlation of 209-gene RCA-MERFISH (after optimization) with bulk RNA-seq from the liver. The correlation is similar to MERFISH without RCA.

2 Figure S2: Measuring spatial patterns of the zonal marker gene expression with RCA-MERFISH, related to Figure 2.
A. Spatial distribution of the expression level of the established zonal (pericentral) gene Cyp1a2 in the RCA-MERFISH assay. The x and y axis are in microns and the color represents per-cell gene expression, in counts-per-thousand.
B. As in A, but for the established zonal (periportal) gene *Aldh1b1*.
C. As in A, but for the established zonal (pericentral) gene *Aldh3a2*.
D. Spatial distribution of the expression level of *Cyp1a2*, showing a zoom-in of the boxed region in A. The x and y axis are in microns and the color represents per-cell gene expression, in counts-per-thousandE. As in D, but for the zonal gene *Aldh1b1*.
F. As in D, but for the zonal gene *Aldh3a2*.
G. Raw amplicons of *Cyp1a2*, decoded from the RCA-MERFISH data, corresponding to the region shown in D. Each RCA-MERFISH amplicon in each cell is represented by a black dot. This representation of the data visualizes the low density of counts of *Cyp1a2* in some regions and the high density in other regions. The x and y axis are in microns.
H. As in G, but showing amplicons of *Aldh1b1*.
I. As in G, but showing amplicons of *Aldh3a2*.
J. Local density of *Cyp1a2* amplicons (red), *Aldh1b1* amplicons (blue), or *Aldh3a2* amplicons (orange) along the indicated line in G-I. Local density is defined as the total number of amplicons within 50 μm of a point. The data are normalized to facilitate the visualization of genes with different average expression levels. The local density of amplicons varies across two orders of magnitude.
K. The ratio of local densities of the indicated gene pairs, along the indicated line in G-I. The *Aldh1b1*/*Cyp1a2* ratio varies across over three orders-of-magnitude, whereas the *Cyp1a2*/*Aldh3a2* ratio remains near 1. This analysis of zonal marker gene expression provides a quantitative assessment of the detection specificity of RCA-MERFISH. Genes with known periportal and pericentral specificity are indeed enriched in periportal and pericentral regions of the tissue, respectively. The RCA-MERFISH amplicon count numbers of these zonal markers are ~100-fold higher in the appropriate spatial zone compared to zones that are not expected to express the markers, demonstrating a high detection specificity for the RCA-MERFISH measurements in tissue.

3 Figure S3: Spatial organization and marker gene expression of different transcriptionally-defined hepatocyte and non-hepatocyte cell types, related to Figure 2.
A. Mean expression of marker genes in non-hepatocyte cell types. Expression is normalized to the cell type with the highest mean expression. The expression levels of cell-type marker genes are much higher in their specific cell types than in other cell types (generally 20–50 folder higher, presumably depending in part on the actual expression specificity of these markers in their respective cell types). Endo: endothelial cell; Fibro: fibroblast.
B. Locations of hepatocyte subtypes and non-hepatocyte cell types in unperturbed liver tissue, as measured by RCA-MERFISH

4 Figure S4: Imaging data processing pipeline and deep learning model architecture, related to Figure 2.
A. Diagram of data processing pipeline. Each panel of measured perturbation barcodes, endogenous RNAs, or morphological data (proteins or RNAs) is collected back-to-back in the same experiment and then processed in parallel. The RCA-MERFISH data (perturbation barcodes and endogenous RNAs) are processed by first registering to common fiducials across multiple rounds, then decoding the identity of individual molecules. The molecules are then filtered using machine learning on features of molecules (mean intensity, size, variance, difference between mean on- and off-bit intensity), to obtain a final 5% false positive rate, measured by the probability of decoding to a blank, invalid barcode. In parallel, the polyA and Na+/K+ ATPase channels of the morphological data are used to segment cells, which are then merged to eliminate duplicates of the same cells segmented in multiple fields of view. The cell segmentations are used to assign decoded RNA molecules to individual cells for quantification, and then the morphological channels are used with the segmentation mask to export the final images and per-gene quantification of expression for each cell.
B. Diagram of final annotated data matrix combining all features.
C. Data preparation for morphological analysis. (Left) The input image for cell segmentation of an example field of view (Blue: polyA RNA. Green: Na+/K+ ATPase). These two channels are fed into a custom CellPose model. (Mid-left) The segmented cell masks, from the CellPose model. (Mid-right) Crop of the single-cell mask for an example cell. A small window containing the cell is cropped out of the field of view. The pixels out of the selected segmentation mask are set to zero. (Right) The images of different staining channels of the cropped cell.
D. High-level diagram of VQ-VAE network across all channels. An input image is put into an artificial neural network that attempts to reconstruct the same image after passing the image through a low-dimensional bottleneck. In this case, two separate representations are created (top- and bottom-level) that attempt to capture different scales of features in the image. The bottom representation is formed first through one network, then further compressed to form a top representation with a separate autoencoder. The two representations are then concatenated and passed through a final network to reconstruct the original image.
E. Detail of VQ-VAE network for each individual channel, trained simultaneously. A separate embedding is created for each morphological channel at the same time, using a mean squared error (MSE) loss to determine the accuracy of reconstruction. For each morphological channel, the embedding is used in a classification task to predict the identity of the protein or RNA being represented. The embeddings for each morphological channel are then concatenated and used to predict higher-level information about the type or state of each cell. When added to the overall training loss, these auxiliary predictive tasks are intended to constrain the representations that are formed by the VQ-VAE networks to capture salient features that discriminate different morphological channels and cell types or states.
F. The change of training and cross-validation losses at different epochs during the training of the VQ-VAE model. The total loss is the sum of the reconstruction loss, the VQ-VAE latent loss, the protein channel classification loss, and the condition classification loss. The training process minimizes the total loss by gradient descent to simultaneously achieve multiple objectives defined by each loss term. The reconstruction loss ensures the latent representation retains enough information to reconstruct the input images. The VQ-VAE latent loss regularizes the complexity of the latent representation. The protein channel/condition classification loss enforces the latent vector to extract biologically meaningful features that distinguish different protein channels/experimental conditions. The cross-validation losses are lower or comparable to the training losses during the training, suggesting the absence of significant overfitting to the training dataset. 85% of data points are used to calculate the training loss, and 15% are used to calculate the cross-validation loss.

5 Figure S5: Analysis of image features from deep learning embedding, related to Figure 2.
A. Diagram of transformation of individual imaging channels into cell by feature representations. Each image channel for each cell is reduced to 512-dimensional vector. Here, we consider each dimension a feature.
B. Heatmap of average feature weights across cells for different imaging channels, with all features shown (left) or only features with high weight scores (high signals) shown (right).
C. (Left) Heatmap of the pairwise correlation between high-signal features across image embeddings. (Right) This heatmap is reordered through hierarchical clustering to reveal features that correlate strongly. Nine classes of features are manually identified and visualized. Each class of features captures similar spatial patterns. Cells with high weight scores of features from several example classes are displayed, including classes ii – cells with two nuclei, class iii – signal enriched at cell membrane, class iv – signals showing relatively diffuse expression, class viii – signals showing locally concentrated, punctate expression, and class ix – noise.
D. Examples of protein channel images that have high values for feature 266 (locally concentrated, punctate expression) from Class viii. This shows that the same protein/RNA feature measures similar spatial patterns across different protein/RNA channels and cells.
E. The spatial distribution of feature 266 in the Perilipin protein channel is illustrated for samples under fasted, high fat diet (HFD), and *ad lib* conditions. Cells displaying high Perilipin 266 values contain high amounts of concentrated perilipin clusters. Notably, pre-normalized values for the Perilipin 266 feature are considerably higher in the HFD sample.

6 Figure S6: Changes and dynamic patterns in gene expression and morphology with physiological state, related to Figure 2.
A. UMAP of individual cells measured by 10X Flex from mice either with *ad lib* diet, overnight fasting, or 1-month high fat diet (HFD), colored by condition (left) or cell-type and subtype identity (right).
B. Genome-wide imputed gene expression of individual hepatocytes (colored by subtype on left). Cells are sorted by periportal gene score, and genes are sorted by correlation with periportal gene score across cells. PP: Periportal; PC: Pericentral. The colors are separately scaled for each gene, considering the maximum and minimum expression of each gene across the population of cells.
C. (top) Spatial organization of hepatocyte subtypes in sections from the indicated condition; (bottom) spatial distribution of lipid biosynthesis gene expression scores, defined as the average normalized expression of a canonical set of lipid and cholesterol synthesis genes (STAR Methods).
D. Zooms showing the spatial organization of hepatocyte subtypes and lipid biosynthesis gene expression with finer resolution.
E. Mean lipid biosynthesis score across hepatocyte subtypes (ordered by the zonation score) under three different physiological conditions (*ad lib*, fasted and HFD).
F. Examples of calreticulin morphology in cells under *ad lib* or HFD conditions.
G. Examples of pS6RP morphology in cells under *ad lib* or HFD conditions.

7 Figure S7: Additional Perturb-seq and Perturb-Multi analyses, related to Figure 3.
A. Diagram of K562-CRISPRi Perturb-seq validation experimentB. Pie chart of the number of called sgRNAs per cell.
C. Histogram representing *ENO1* expression in cells with an sgRNA targeting *ENO1* or in cells with control sgRNAs.
D. Histogram representing on-target knockdown and off-target knockdown, combining all gene targets in the experiment. On-target knockdown is defined as the average (pseudobulk) expression of the target gene in cells with each corresponding sgRNA, relative to cells with control sgRNAs. Off-target knockdown is defined as the expression of each of other genes targeted in the experiment (not targeted in cells with a given called sgRNA), relative to expression of those genes in cells with control sgRNAs.
E. Heat map representation of average expression of each of the indicated genes in cells with each of the indicated sgRNAs, relative to expression of those genes in cells with control sgRNAs.
F. Unbiased sampling of cells with control sgRNAs and sgRNAs targeting *Gapdh*. The fluorescence micrographs show anti-GAPDH and polyA FISH channels.
G. Histogram comparing anti-Gapdh intensity in called cells with a control sgRNA and called cells with *Gapdh*-targeting sgRNAs, from the imaging dataset.
H. Pie charts showing the number of targeting (left) and control (right) sgRNAs that caused a significant transcriptional phenotype, as measured by a Holm-Šídák-corrected energy distance permutation test (p < 0.05), in the imaging dataset. 84/402 targeting sgRNAs and 3/50 non-targeting sgRNAs have significant phenotypes.
I. Scatterplot comparing the energy distance vs control cells for each knockout in the imaging and Perturb-seq datasets.
J. Histogram representing the number of differentially expressed genes for each perturbation, from the sequencing experiment. Significant differential gene expression is determined by Benjamini-Hochberg-corrected, Mann-Whitney p < 0.05, versus cells with control sgRNAs.
K. Histogram representing the number of imaging channels (proteins or RNAs) exhibiting differentially intense signals for each perturbation, from the imaging experiment. Significant differential intensity is determined by Benjamini-Hochberg-corrected, Mann-Whitney p < 0.05, versus cells with control sgRNAs.

8 Table S1: Fluorescent readout oligonucleotide bits and RCA-MERFISH padlock probe sequences, related to Figures 1 and 3)○ The sequences and colors of the fluorescent readout oligonucleotide bits used for all RCA-MERFISH imaging.○ The targets and sequences of the padlock probes used for the mRNA measurements in RCA-MERFISH.○ The targets and sequences of the padlock probes used for the perturbation barcode measurements in RCA-MERFISH.

9 Table S2: Oligo-Antibody + Abundant RNA Panel, related to Figures 2 and 3○ The antibodies used for multiplexed oligo-antibody immunofluorescence, as well as the abundant RNAs targeted with sequential FISH.

10 Table S3: Abundant RNA Probe Sequences, related to Figures 2 and 3○ Sequences of the oligonucleotide probes used for FISH targeting abundant RNAs.

11 Table S4: RCA-MERFISH Imaging Experiment Rounds, related to Figures 2 and 3○ Description of the imaging rounds in the RCA-MERFISH experiments conducted in WT animals.○ Description of the imaging rounds in the RCA-MERFISH experiments conducted in genetic mosaic animals.

12 Table S5: Liver Perturbation Library, related to Figure 3○ Targets, sgRNA sequences, and barcode sequences used to generate the genetic mosaic livers that we analyzed with Perturb-Multi.

13 Table S6: Fixed Cell Perturb-seq sgRNA Probes, related to Figure 3○ Sequences of sgRNA-targeting probes used in fixed-cell Perturb-seq.

14 Method S1: Bench Protocol for RCA-MERFISH probe preparation and sample preparation, related to Figures 1 and 3○ Step-by-step bench protocol for RCA-MERFISH probe preparation and imaging sample preparation.

## Figures and Tables

**Table:** KEY RESOURCES TABLE

REAGENT or RESOURCE	SOURCE	IDENTIFIER
**Antibodies**		
anti-Calreticulin	Abcam	ab271865
anti-CathB	CST	D1C7YBF
anti-Gapdh	CST	14C10BF
anti-LC3b	CST	3868BF
anti-M6pr	Abcam	ab226090
anti-Na+/K+	Abcam	ab167390
anti-Perilipin	Proteintech	15294–1-AP
anti-pS6RP	CST	2211BF
anti-Rab7	Abcam	ab214806
anti-SNAP23	Abcam	ab271890
anti-SQSTM1	Abcam	ab227992
anti-Tomm20	Abcam	ab232589
anti-Tomm70	Proteintech	14528–1-AP
anti-Vimentin	Abcam	ab193555
		
**Bacterial and virus strains**		
Stellar Competent Cells	Takara	Cat# 636766
MegaX Electrocompetent Cells	ThermoFisher	Cat# C640003
		
**Chemicals, peptides, and recombinant proteins**		
Formamide	Ambion	Cat# AM9342
20xSSC	Ambion	Cat# AM9763
Triton X-100	Sigma	Cat# T8787
Glucose oxidase	Sigma	Cat# G2133
Phusion® Hot Start Flex 2X Master Mix	New England Biolabs	Cat# M0536
Maxima H Minus Reverse Transcriptase	ThermoFisher	Cat# EP0752
dNTP mix	ThermoFisher	Cat# R1121
32% Paraformaldehyde	Electron Microscopy Sciences	Cat# 15714S
RNase inhibitor, Murine	New England Biolabs	Cat# M0314
1M Tris, pH 8	ThermoFisher	Cat# 15568025
Catalase	Sigma	Cat# C3155
6-hydroxy-2,5,7,8-tetramethylchroman-2-carboxylic acid (Trolox)	Sigma	Cat# 238813
Tris(2-carboxyethyl)phosphine (TCEP) HCl	GoldBio	Cat# TCEP1
Hoescht 33,342, Trihydrochloride, Trihydrate	ThermoFisher	Cat# H3570
Lipopolysaccharides from *E. coli* O 111:B4	Sigma	Cat# L4391
Yeast tRNA	ThermoFisher	Cat# AM7119
Dextran sulfate	Sigma	Cat# S4030
Ethanol	Decon Labs	Cat# V1016
SDS	ThermoFisher	Cat# 15553027
Proteinase K	New England Biolabs	Cat# P8107S
Ethylene carbonate	Sigma	Cat# 676802–1L
Glucose	Sigma	Cat# 49139
Water	Ambion	Cat# AM9932
T4 Ligase	NEB	Cat# M0202S
T4 Ligase (High Concentration)	NEB	Cat# M0202T
BstXI	NEB	Cat# R0113S
BamHI	NEB	Cat# R0136S
Fugene HD	Promega	Cat# E2311
ViralBoost	Alstem	Cat# VB100
Lenti-X™ Concentrator	Takara	Cat# 631232
BSA	Ambion	Cat# AM2616
Sheared Salmon Sperm DNA	Ambion	Cat# AM9680
SUPERase·In RNase Inhibitor	Ambion	Cat# AM2694
TE pH 9	GeneMed	Cat# 10–0046
BS(PEG)9	ThermoFisher	Cat# 21582
Melphalan	SelleckChem	Cat# S8266
MOPS	Sigma	Cat# 69947
40% Acrylamide/Bis Solution, 19:1	Bio-Rad	Cat# 1610144
TEMED	Sigma	Cat# T7024
Ammonium persulfate	Sigma	Cat# A3678
PEG35000	Sigma	Cat# 94646
Tween 20	Sigma	Cat# P9416
SplintR Ligase	NEB	Cat# M0375S
NxGen phi29 DNA Polymerase	Lucigen	Cat# 30221–3
dNTP Mix	NEB	Cat# N0447S
Aminoallyl-dUTP	ThermoFisher	Cat# R1101
BccI	NEB	Cat# R0704S
BciVI	NEB	Cat# R0596S
NaCl	Ambion	Cat# AM9759
MgCl2	Ambion	Cat# AM9530G
TE	Ambion	Cat# AM9849
Invitrogen™ SiteClick™ Antibody Azido Modification Kit	ThermoFisher	Cat# S10900
sulfo-Cyanine3 NHS ester	Lumiprobe	Cat# 61320
sulfo-Cyanine5 NHS ester	Lumiprobe	Cat# 63320
sulfo-Cyanine7 NHS ester	Lumiprobe	Cat# 65320
		
**Critical commercial assays**		
Chromium Fixed RNA Kit, Mouse Transcriptome, 4rxns x 4 BC	10x Genomics	Cat#: 1000496
		
**Deposited data**		
Raw and analyzed scRNA-seq data	This Study	GEO: GSE275483
Cell Images	This Study	FigShare (available upon publication or request for review purposes)
		
**Experimental models: Cell lines**		
K562-CRISPRi Cells	Previous Study	Gilbert *et al*, 2014
293T/17	ATCC	CRL-11268
		
**Experimental models: Organisms/strains**		
B6J.129(B6N)-Gt(ROSA)26Sor^tm1(CAG-cas9*,-EGFP)Fezh^/J	JAX	Strain #: 026179
C57BL/6J	JAX	Strain #: 000664
		
**Oligonucleotides**		
Readout Probes	Integrated DNA Technologies	See Table S1
Abundant RNA FISH Probes	Integrated DNA Technologies	See Table S3
RCA-MERFISH Padlock Probe Library (mRNA)	Twist Bioscience	See Table S1
RCA-MERFISH Padlock Probe Library (Perturbation Barcodes)	Twist Bioscience	See Table S1
RCA Primer: TCTTCACCCGGGGCAGCTGAA*G*T	Integrated DNA Technologies	N/A
polyA FISH Probe: /5Acryd/TTGAGTGGATGGAGTGTAATT+TT+TT + TT + TT + TT + TT + TT + TT + TT + T	Integrated DNA Technologies	N/A
Liver Perturbation Library	Integrated DNA Technologies	See Table S5
Perturb-seq sgRNA Probes	Integrated DNA Technologies	See Table S6
		
**Recombinant DNA**		
pVV1	This study	Deposit to Addgene in progress
		
**Software and algorithms**		
CellRanger	10x Genomics	https://www.10xgenomics.com/software
Custom Analysis Software	This Paper	https://github.com/weallen/InVivoMultimodalPerturbation
Scanpy	Ref. ^114^	https://github.com/scverse/scanpy
Anndata	Ref. ^115^	https://github.com/scverse/anndata
scPerturb	Ref. ^66^	https://github.com/sanderlab/scPerturb
MERlin	Ref. ^99^	https://github.com/ZhuangLab/MERlin
Harmony	Ref. ^116^	https://github.com/slowkow/harmonypy
CellPose	Ref. ^117^	https://github.com/MouseLand/cellpose

## Highlights:

- Perturb-Multi merges imaging with scRNA-seq for pooled CRISPR screens in tissue
- RCA-MERFISH captures *in situ* genotype and multiplex RNA-protein phenotypes
- Fixed-cell Perturb-seq enables *in vivo* CRISPR screens with intact transcriptomes
- Multimodal readouts provide insights into gene control of various liver functions
