# Supplementary material for: Perturb-Multimodal: a platform for pooled genetic screens with sequencing and imaging in intact mammalian tissue
Source: Cell. Author manuscript; Available in PMC 2025 Aug 6. (PMC12324982; doi:10.1016/j.cell.2025.05.022)
Supplement: 1 — Figure S1: Development and optimization of the RCA-MERFISH protocol, related to Figure 1. A. Phi29 used in RCA degrades ssDNA FISH probes. Left: Single-molecule FISH signal in U-2 OS cells without Phi29 treatment. Center: Single-molecule FISH signal in U-2 OS cells pre-treated with Phi29 at 37oC for 1 hr before FISH staining. Right: Single-molecule FISH signal in U-2 OS cells treated with Phi29 at 37oC for 1 hr after FISH staining B. Quantification of the effect of Phi29 on ssDNA FISH probes from (A), in spots/cell. C. Quantification of the effect of Phi29 on ssDNA FISH probes from (A), in intensity/spot. D. Dextran sulfate (crowding agent) inclusion in hybridization buffer inhibits Phi29 enzymatic activity. Left: RCA-MERFISH signal with padlock probe against GFP in U-2 OS cells not expressing GFP, detected by readout probes complementary to readout sequences on the padlock. Center: RCA-MERFISH signal with padlock probe against GFP in U-2 OS cells expressing GFP, with dextran sulfate in the hybridization buffer. Right: RCA-MERFISH signal with padlock probe against GFP in U-2 OS cells expressing GFP, without dextran sulfate in the hybridization buffer. E. Optimization of alternative crowding agents to dextran sulfate. Multiple additives to hybridization mixture, staining U-2 OS cells expressing either GFP or mCherry with a single probe against GFP, in terms of number of spots per cell, distinguishing GFP+ (signal) and mCherry+ (background) cells. Peg8k = Poly(ethylene glycol) average mol wt 8,000, Peg35k = Poly(ethylene glycol) average mol wt 35,000, Dextran = unsulfonated dextran; all are added to the hybridization solution so the final w/v is at the indicated percent. F. RCA amplicons of RCA-MERFISH probe against GFP in U-2 OS cells expressing either GFP or mCherry. These data are from the PEG35K 5% condition. There are many more GFP amplicons in GFP-expressing cells than there are in mCherry-expressing cells, indicating the specificity of RCA-MERFISH. The specific [file NIHMS2091173-supplement-1.pdf]

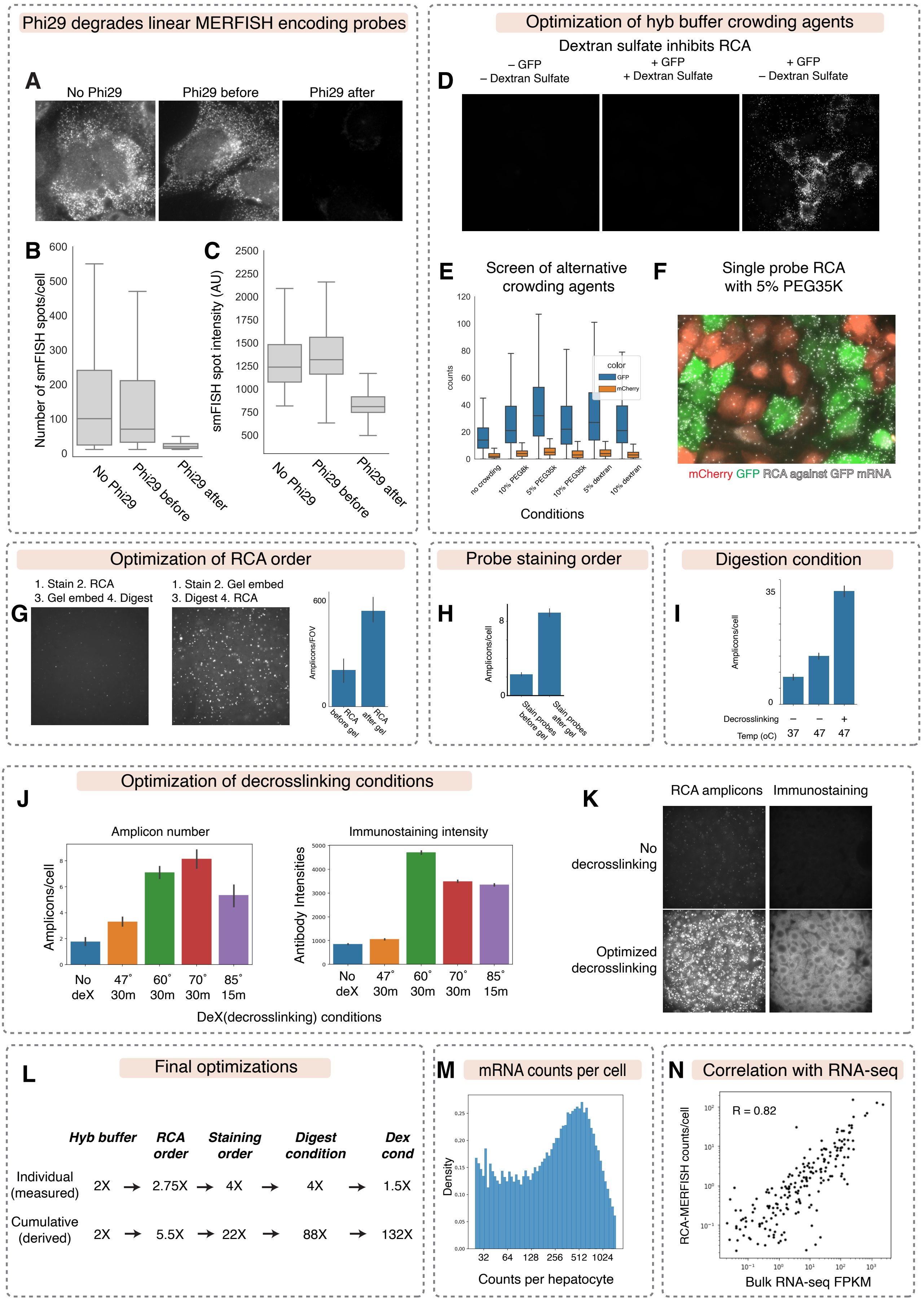
Phi29 degrades linear MERFISH encoding probes
A
No Phi29
Phi29 before
Phi29 after
B
Number of smFISH spots/cell
C
smFISH spot intensity (AU)
No Phi29
Phi29 before
Phi29 after
Optimization of hyb buffer crowding agents
Dextran sulfate inhibits RCA
D
– GFP
– Dextran Sulfate
+ GFP
+ Dextran Sulfate
+ GFP
– Dextran Sulfate
E
Screen of alternative crowding agents
counts
color
GFP
mCherry
no crowding
10% PEG8k
5% PEG35k
10% PEG35k
5% dextran
10% dextran
Conditions
F
Single probe RCA with 5% PEG35K
mCherry GFP RCA against GFP mRNA
Optimization of RCA order
G
1. Stain 2. RCA
3. Gel embed 4. Digest
1. Stain 2. Gel embed
3. Digest 4. RCA
Amplicons/FOV
RCA before gel
RCA after gel
Probe staining order
H
Amplicons/cell
Stain probes before gel
Stain probes after gel
Digestion condition
I
Amplicons/cell
Decrosslinking – – +
Temp (oC) 37 47 47
Optimization of decrosslinking conditions
J
Amplicon number
Amplicons/cell
Immunostaining intensity
Antibody Intensities
No deX
47˚ 30m
60˚ 30m
70˚ 30m
85˚ 15m
DeX(decrosslinking) conditions
K
RCA amplicons
Immunostaining
No decrosslinking
Optimized decrosslinking
L
Final optimizations
Hyb buffer
RCA order
Staining order
Digest condition
Dex cond
Individual (measured) 2X → 2.75X → 4X → 4X → 1.5X
Cumulative (derived) 2X → 5.5X → 22X → 88X → 132X
M
mRNA counts per cell
Density
Counts per hepatocyte
N
Correlation with RNA-seq
R = 0.82
RCA-MERFISH counts/cell
Bulk RNA-seq FPKM
