# Supplementary material for: Perturb-Multimodal: a platform for pooled genetic screens with sequencing and imaging in intact mammalian tissue
Source: Cell. Author manuscript; Available in PMC 2025 Aug 6. (PMC12324982; doi:10.1016/j.cell.2025.05.022)
Supplement: 2 — Figure S2: Measuring spatial patterns of the zonal marker gene expression with RCA-MERFISH, related to Figure 2. A. Spatial distribution of the expression level of the established zonal (pericentral) gene Cyp1a2 in the RCA-MERFISH assay. The x and y axis are in microns and the color represents per-cell gene expression, in counts-per-thousand. B. As in A, but for the established zonal (periportal) gene Aldh1b1. C. As in A, but for the established zonal (pericentral) gene Aldh3a2. D. Spatial distribution of the expression level of Cyp1a2, showing a zoom-in of the boxed region in A. The x and y axis are in microns and the color represents per-cell gene expression, in counts-per-thousandE. As in D, but for the zonal gene Aldh1b1. F. As in D, but for the zonal gene Aldh3a2. G. Raw amplicons of Cyp1a2, decoded from the RCA-MERFISH data, corresponding to the region shown in D. Each RCA-MERFISH amplicon in each cell is represented by a black dot. This representation of the data visualizes the low density of counts of Cyp1a2 in some regions and the high density in other regions. The x and y axis are in microns. H. As in G, but showing amplicons of Aldh1b1. I. As in G, but showing amplicons of Aldh3a2. J. Local density of Cyp1a2 amplicons (red), Aldh1b1 amplicons (blue), or Aldh3a2 amplicons (orange) along the indicated line in G-I. Local density is defined as the total number of amplicons within 50 μm of a point. The data are normalized to facilitate the visualization of genes with different average expression levels. The local density of amplicons varies across two orders of magnitude. K. The ratio of local densities of the indicated gene pairs, along the indicated line in G-I. The Aldh1b1/Cyp1a2 ratio varies across over three orders-of-magnitude, whereas the Cyp1a2/Aldh3a2 ratio remains near 1. This analysis of zonal marker gene expression provides a quantitative assessment of the detection specificity of RCA-MERFISH. Genes with known periportal and pericentral specificity [file NIHMS2091173-supplement-2.pdf]

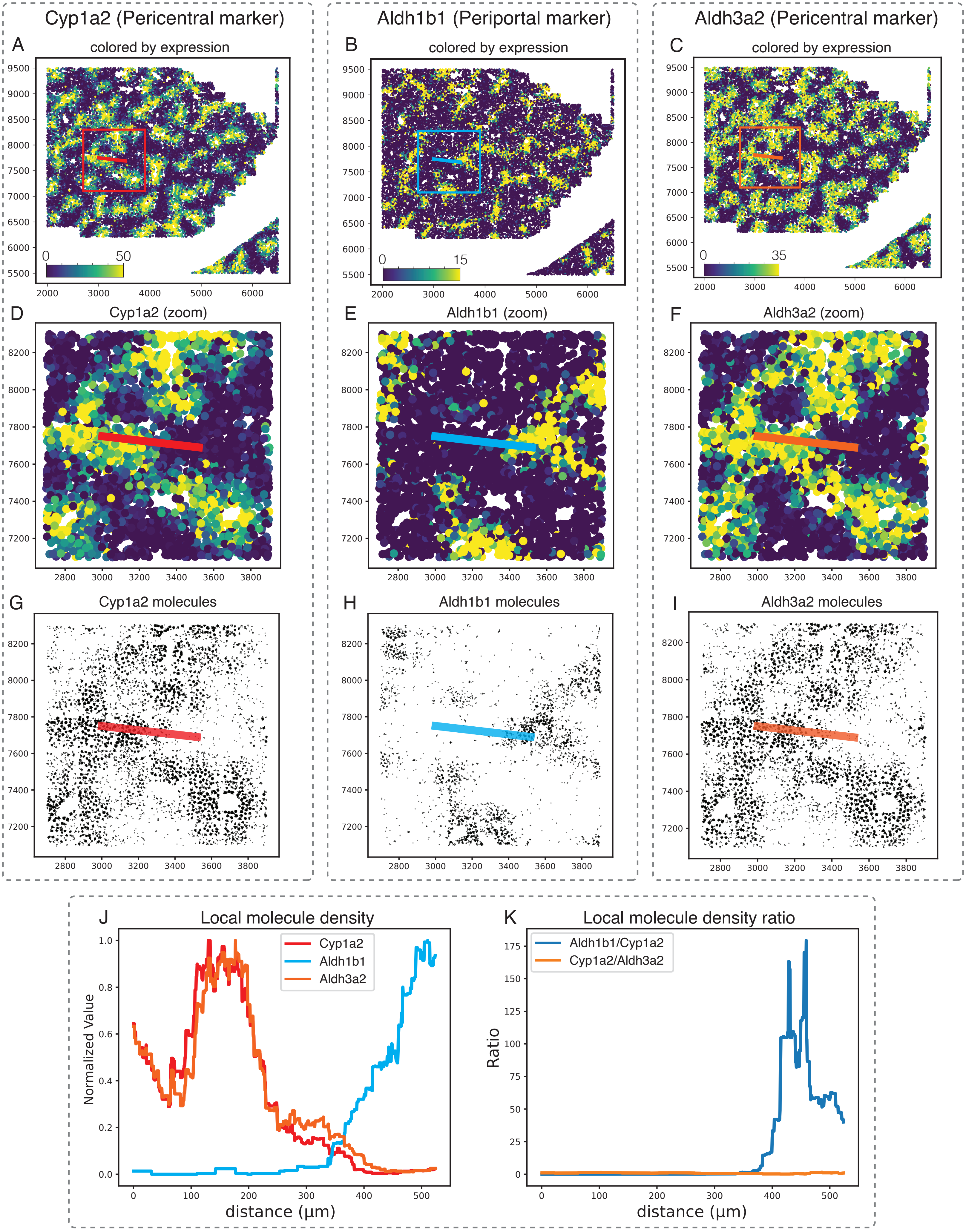
Cyp1a2 (Pericentral marker)
Aldh1b1 (Periportal marker)
Aldh3a2 (Pericentral marker)
A
B
C
colored by expression
D
Cyp1a2 (zoom)
E
Aldh1b1 (zoom)
F
Aldh3a2 (zoom)
G
Cyp1a2 molecules
H
Aldh1b1 molecules
I
Aldh3a2 molecules
J
Local molecule density
Normalized Value
distance (µm)
Cyp1a2
Aldh1b1
Aldh3a2
K
Local molecule density ratio
Ratio
distance (µm)
Aldh1b1/Cyp1a2
Cyp1a2/Aldh3a2
