# Supplementary figures and images for: Perturb-Multimodal: a platform for pooled genetic screens with sequencing and imaging in intact mammalian tissue
Source: Cell. Author manuscript; Available in PMC 2025 Aug 6. (PMC12324982; doi:10.1016/j.cell.2025.05.022)

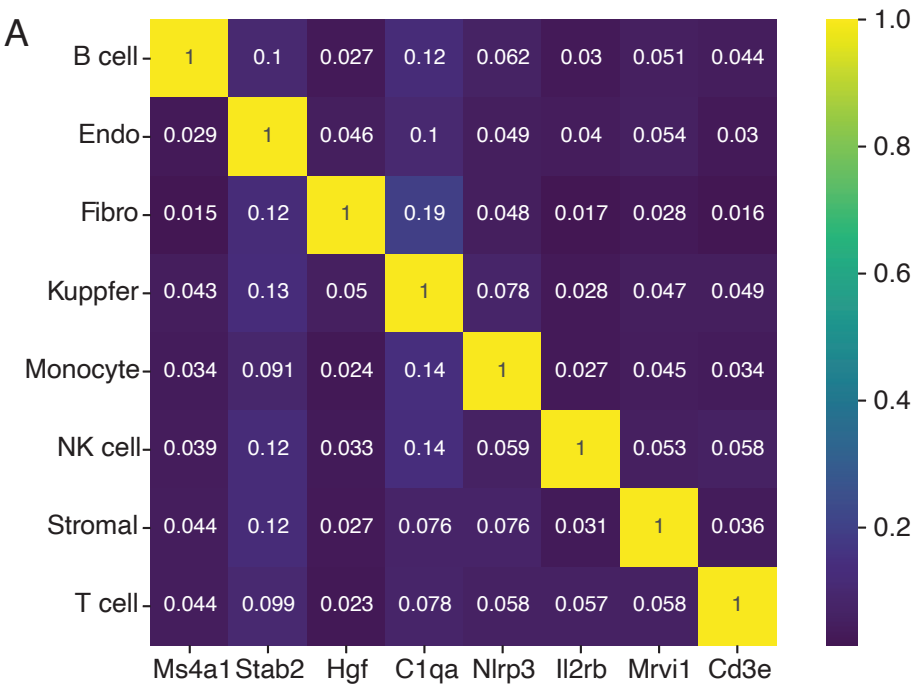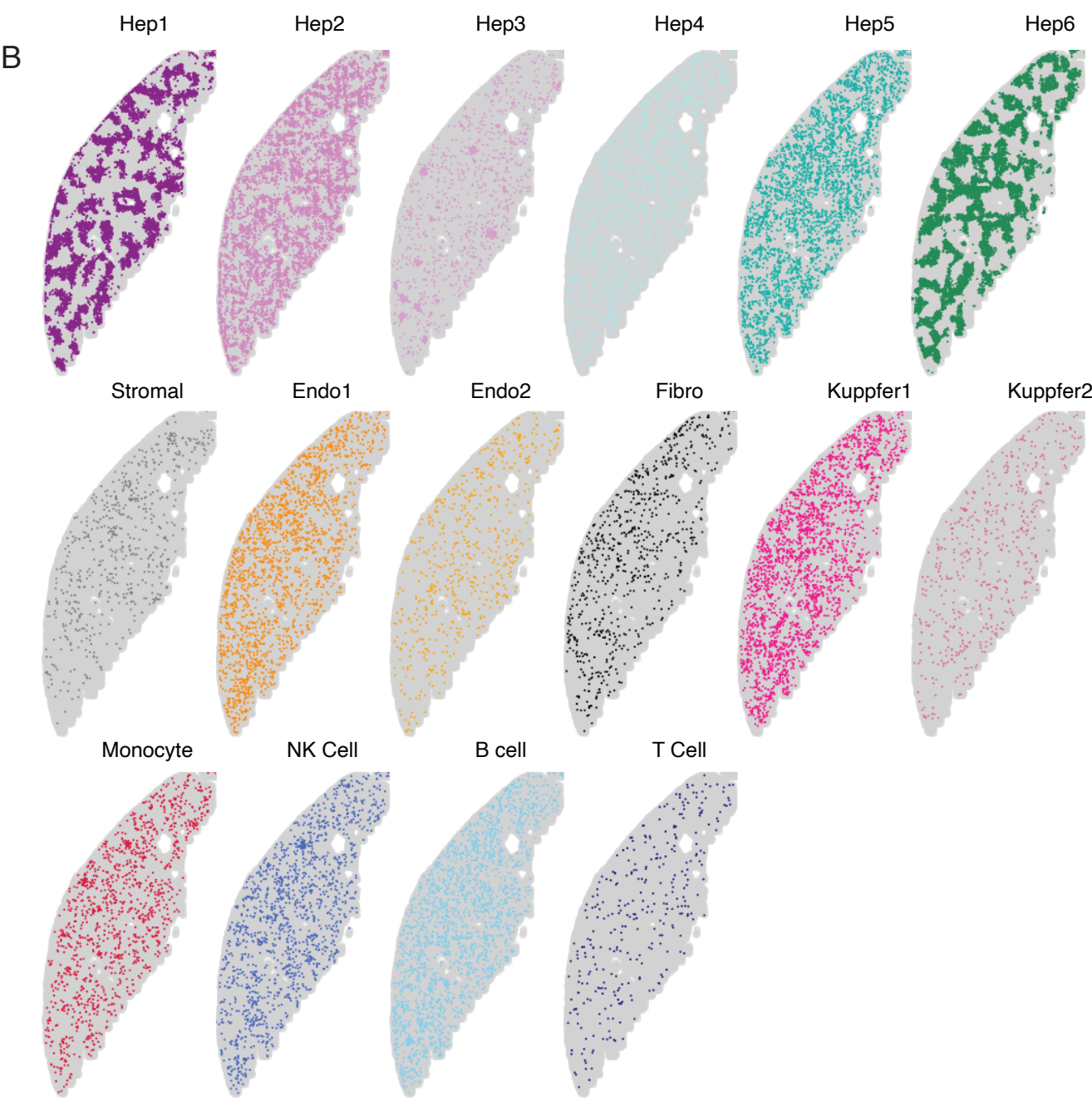

Supplement: 3 — Figure S3: Spatial organization and marker gene expression of different transcriptionally-defined hepatocyte and non-hepatocyte cell types, related to Figure 2. A. Mean expression of marker genes in non-hepatocyte cell types. Expression is normalized to the cell type with the highest mean expression. The expression levels of cell-type marker genes are much higher in their specific cell types than in other cell types (generally 20–50 folder higher, presumably depending in part on the actual expression specificity of these markers in their respective cell types). Endo: endothelial cell; Fibro: fibroblast. B. Locations of hepatocyte subtypes and non-hepatocyte cell types in unperturbed liver tissue, as measured by RCA-MERFISH [file NIHMS2091173-supplement-3.pdf]

**A****Data processing pipeline**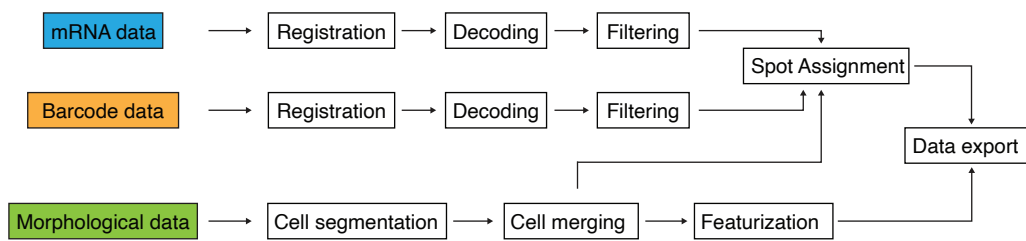**B****Annotated data matrix**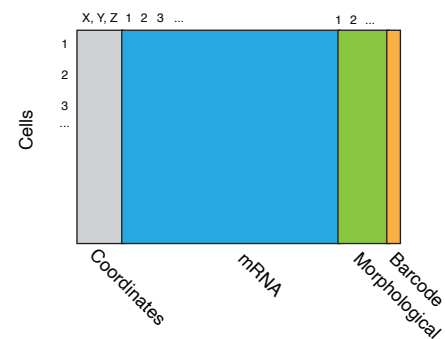**C**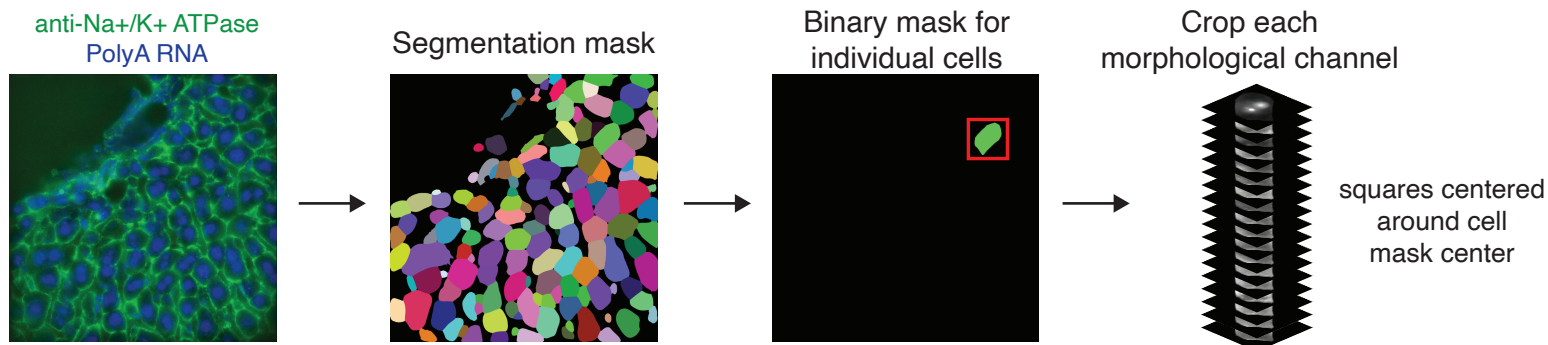**D**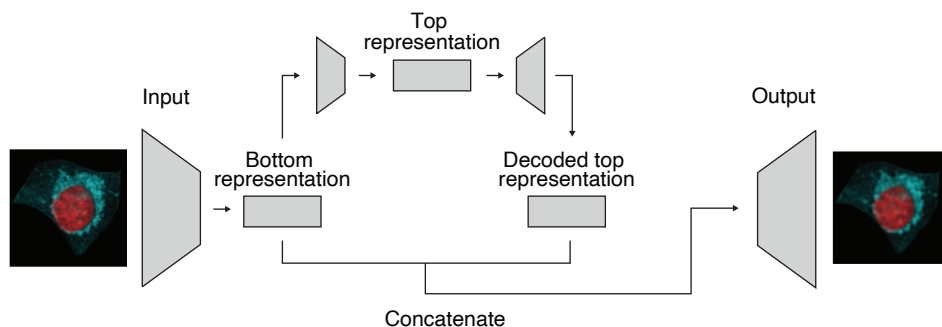**E**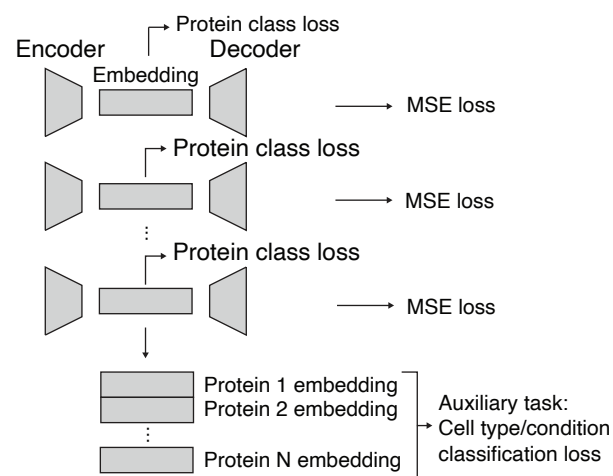**F**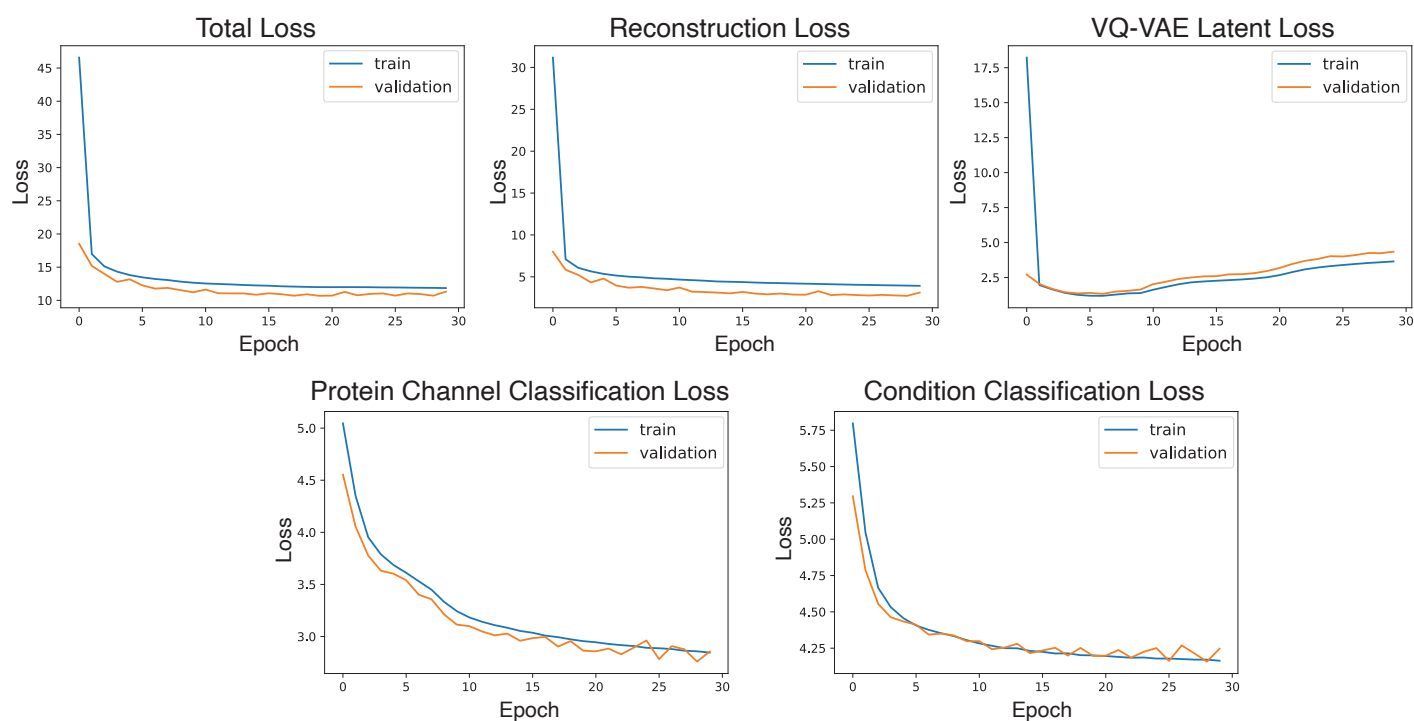

Supplement: 4 — Figure S4: Imaging data processing pipeline and deep learning model architecture, related to Figure 2. A. Diagram of data processing pipeline. Each panel of measured perturbation barcodes, endogenous RNAs, or morphological data (proteins or RNAs) is collected back-to-back in the same experiment and then processed in parallel. The RCA-MERFISH data (perturbation barcodes and endogenous RNAs) are processed by first registering to common fiducials across multiple rounds, then decoding the identity of individual molecules. The molecules are then filtered using machine learning on features of molecules (mean intensity, size, variance, difference between mean on- and off-bit intensity), to obtain a final 5% false positive rate, measured by the probability of decoding to a blank, invalid barcode. In parallel, the polyA and Na+/K+ ATPase channels of the morphological data are used to segment cells, which are then merged to eliminate duplicates of the same cells segmented in multiple fields of view. The cell segmentations are used to assign decoded RNA molecules to individual cells for quantification, and then the morphological channels are used with the segmentation mask to export the final images and per-gene quantification of expression for each cell. B. Diagram of final annotated data matrix combining all features. C. Data preparation for morphological analysis. (Left) The input image for cell segmentation of an example field of view (Blue: polyA RNA. Green: Na+/K+ ATPase). These two channels are fed into a custom CellPose model. (Mid-left) The segmented cell masks, from the CellPose model. (Mid-right) Crop of the single-cell mask for an example cell. A small window containing the cell is cropped out of the field of view. The pixels out of the selected segmentation mask are set to zero. (Right) The images of different staining channels of the cropped cell. D. High-level diagram of VQ-VAE network across all channels. An input image is put into an artificial neural network [file NIHMS2091173-supplement-4.pdf]

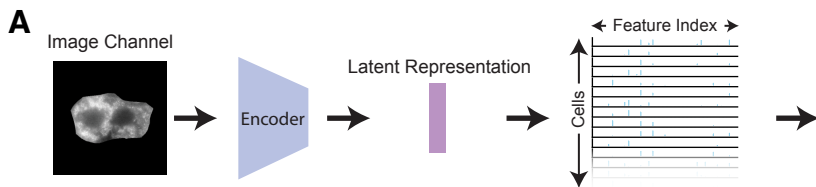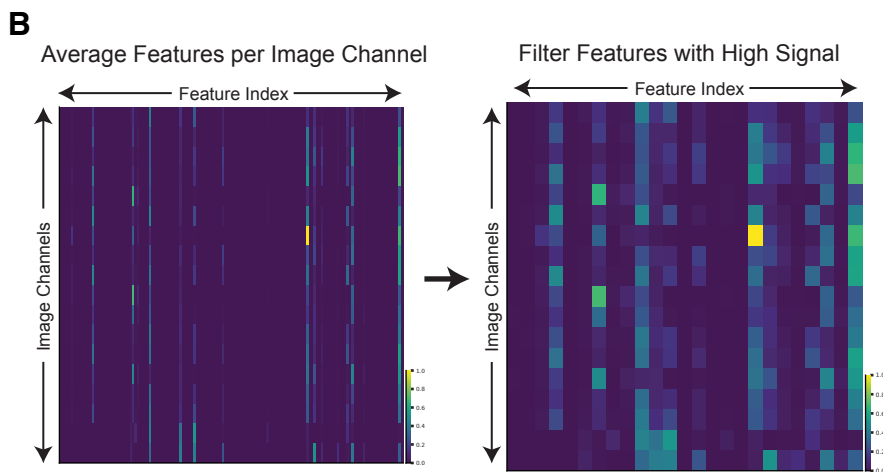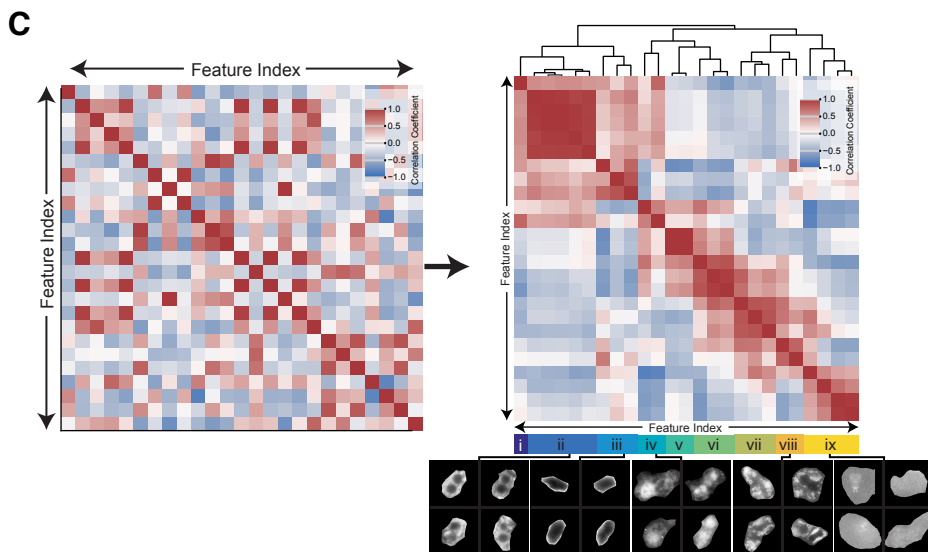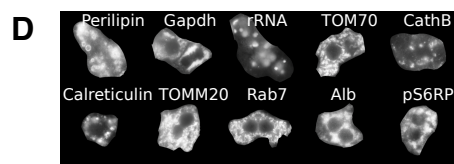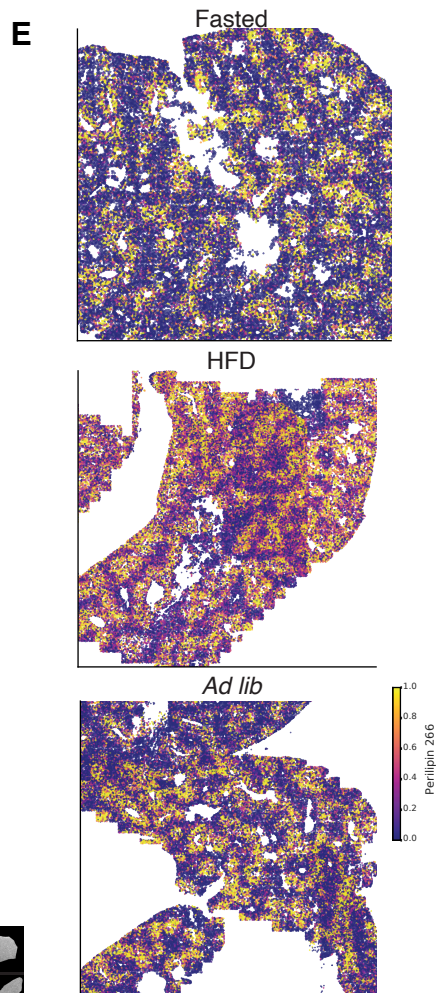

Supplement: 5 — Figure S5: Analysis of image features from deep learning embedding, related to Figure 2. A. Diagram of transformation of individual imaging channels into cell by feature representations. Each image channel for each cell is reduced to 512-dimensional vector. Here, we consider each dimension a feature. B. Heatmap of average feature weights across cells for different imaging channels, with all features shown (left) or only features with high weight scores (high signals) shown (right). C. (Left) Heatmap of the pairwise correlation between high-signal features across image embeddings. (Right) This heatmap is reordered through hierarchical clustering to reveal features that correlate strongly. Nine classes of features are manually identified and visualized. Each class of features captures similar spatial patterns. Cells with high weight scores of features from several example classes are displayed, including classes ii – cells with two nuclei, class iii – signal enriched at cell membrane, class iv – signals showing relatively diffuse expression, class viii – signals showing locally concentrated, punctate expression, and class ix – noise. D. Examples of protein channel images that have high values for feature 266 (locally concentrated, punctate expression) from Class viii. This shows that the same protein/RNA feature measures similar spatial patterns across different protein/RNA channels and cells. E. The spatial distribution of feature 266 in the Perilipin protein channel is illustrated for samples under fasted, high fat diet (HFD), and ad lib conditions. Cells displaying high Perilipin 266 values contain high amounts of concentrated perilipin clusters. Notably, pre-normalized values for the Perilipin 266 feature are considerably higher in the HFD sample. [file NIHMS2091173-supplement-5.pdf]

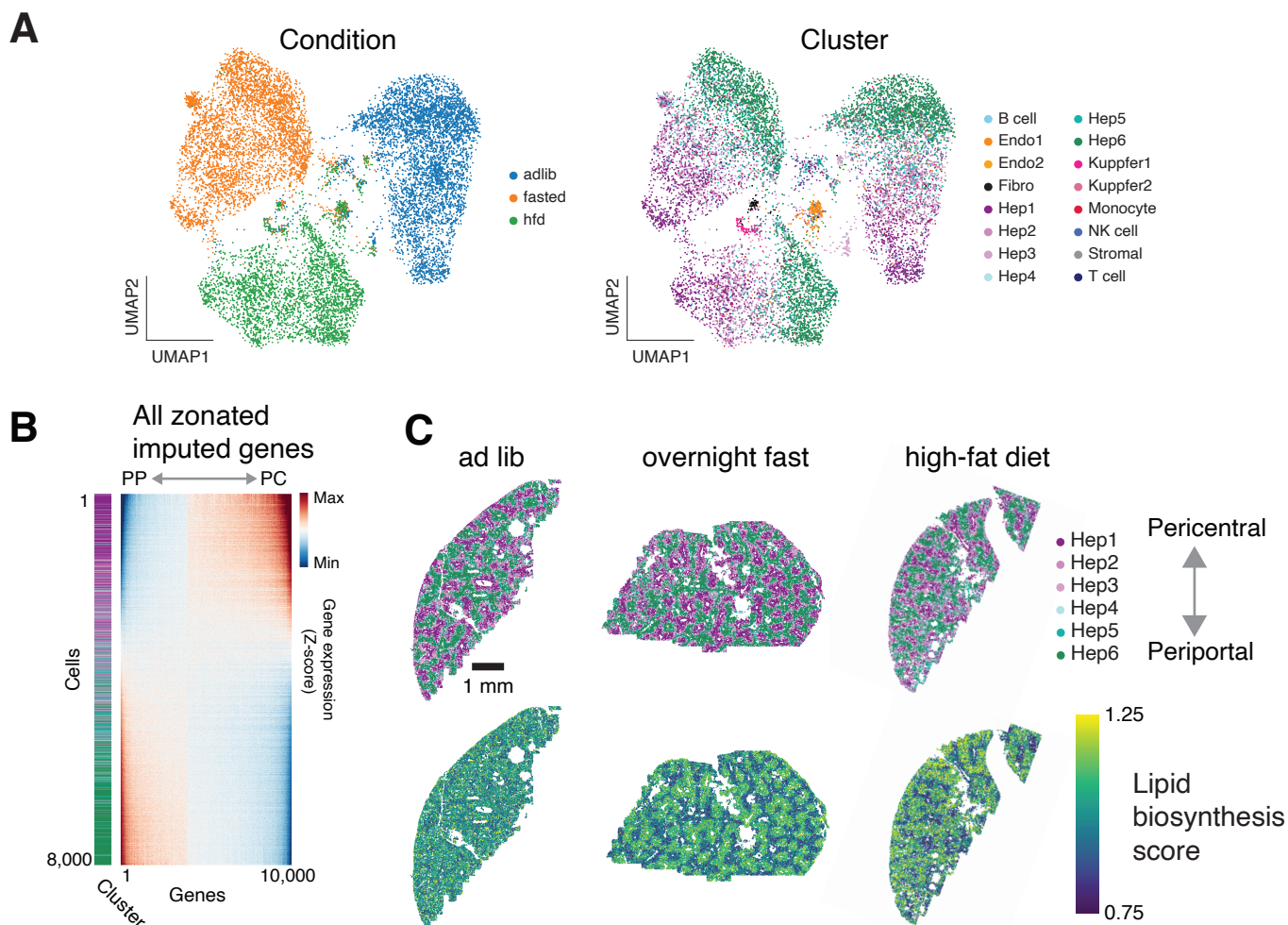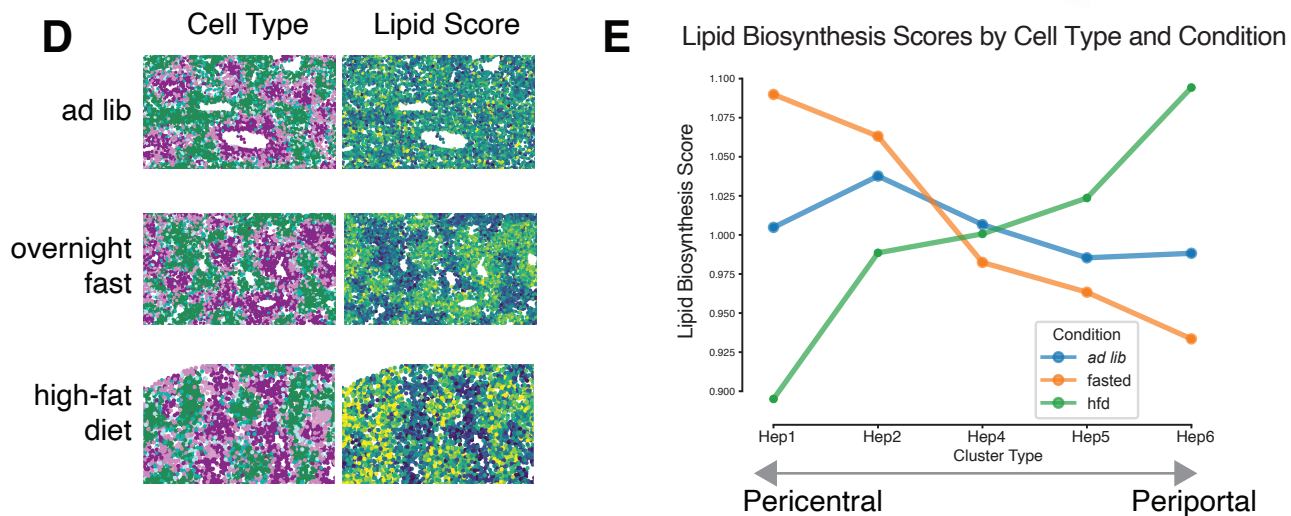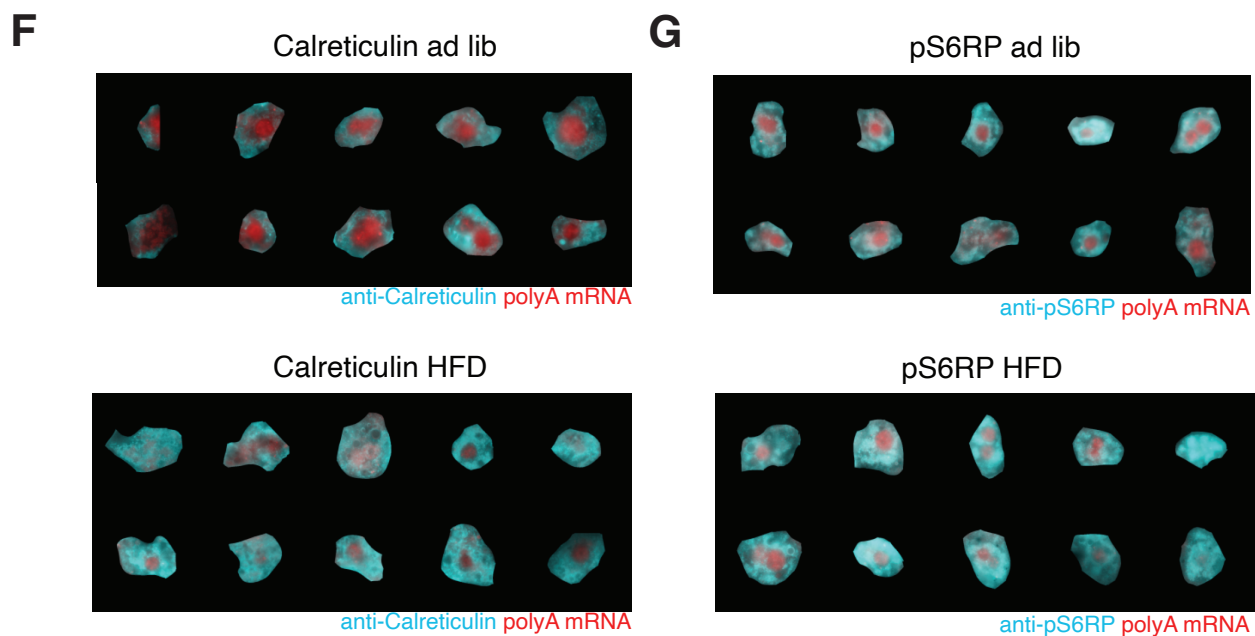

Supplement: 6 — Figure S6: Changes and dynamic patterns in gene expression and morphology with physiological state, related to Figure 2. A. UMAP of individual cells measured by 10X Flex from mice either with ad lib diet, overnight fasting, or 1-month high fat diet (HFD), colored by condition (left) or cell-type and subtype identity (right). B. Genome-wide imputed gene expression of individual hepatocytes (colored by subtype on left). Cells are sorted by periportal gene score, and genes are sorted by correlation with periportal gene score across cells. PP: Periportal; PC: Pericentral. The colors are separately scaled for each gene, considering the maximum and minimum expression of each gene across the population of cells. C. (top) Spatial organization of hepatocyte subtypes in sections from the indicated condition; (bottom) spatial distribution of lipid biosynthesis gene expression scores, defined as the average normalized expression of a canonical set of lipid and cholesterol synthesis genes (STAR Methods). D. Zooms showing the spatial organization of hepatocyte subtypes and lipid biosynthesis gene expression with finer resolution. E. Mean lipid biosynthesis score across hepatocyte subtypes (ordered by the zonation score) under three different physiological conditions (ad lib, fasted and HFD). F. Examples of calreticulin morphology in cells under ad lib or HFD conditions. G. Examples of pS6RP morphology in cells under ad lib or HFD conditions. [file NIHMS2091173-supplement-6.pdf]
