# Supplementary material for: Perturb-Multimodal: a platform for pooled genetic screens with sequencing and imaging in intact mammalian tissue
Source: Cell. Author manuscript; Available in PMC 2025 Aug 6. (PMC12324982; doi:10.1016/j.cell.2025.05.022)
Supplement: 7 — Figure S7: Additional Perturb-seq and Perturb-Multi analyses, related to Figure 3. A. Diagram of K562-CRISPRi Perturb-seq validation experimentB. Pie chart of the number of called sgRNAs per cell. C. Histogram representing ENO1 expression in cells with an sgRNA targeting ENO1 or in cells with control sgRNAs. D. Histogram representing on-target knockdown and off-target knockdown, combining all gene targets in the experiment. On-target knockdown is defined as the average (pseudobulk) expression of the target gene in cells with each corresponding sgRNA, relative to cells with control sgRNAs. Off-target knockdown is defined as the expression of each of other genes targeted in the experiment (not targeted in cells with a given called sgRNA), relative to expression of those genes in cells with control sgRNAs. E. Heat map representation of average expression of each of the indicated genes in cells with each of the indicated sgRNAs, relative to expression of those genes in cells with control sgRNAs. F. Unbiased sampling of cells with control sgRNAs and sgRNAs targeting Gapdh. The fluorescence micrographs show anti-GAPDH and polyA FISH channels. G. Histogram comparing anti-Gapdh intensity in called cells with a control sgRNA and called cells with Gapdh-targeting sgRNAs, from the imaging dataset. H. Pie charts showing the number of targeting (left) and control (right) sgRNAs that caused a significant transcriptional phenotype, as measured by a Holm-Šídák-corrected energy distance permutation test (p < 0.05), in the imaging dataset. 84/402 targeting sgRNAs and 3/50 non-targeting sgRNAs have significant phenotypes. I. Scatterplot comparing the energy distance vs control cells for each knockout in the imaging and Perturb-seq datasets. J. Histogram representing the number of differentially expressed genes for each perturbation, from the sequencing experiment. Significant differential gene expression is determined by Benjamini-Hochberg-corrected, Mann-Whitney p < 0.05, versus cell [file NIHMS2091173-supplement-7.pdf]

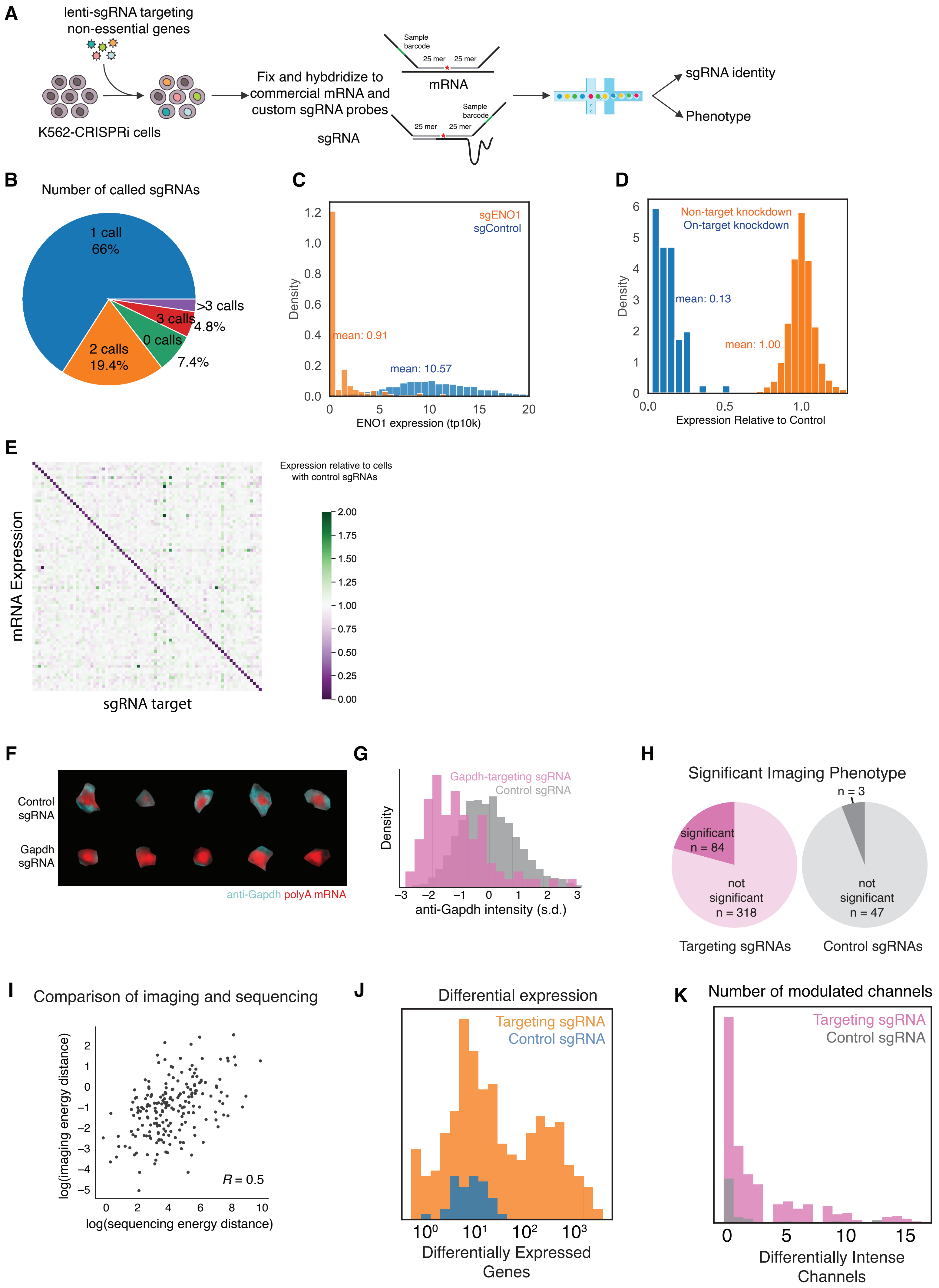
A
lenti-sgRNA targeting non-essential genes
K562-CRISPRi cells
Fix and hybdridize to commercial mRNA and custom sgRNA probes
Sample barcode
25 mer
25 mer
mRNA
sgRNA
sgRNA identity
Phenotype
B
Number of called sgRNAs
1 call 66%
2 calls 19.4%
0 calls 7.4%
3 calls 4.8%
>3 calls
C
sgENO1
sgControl
mean: 0.91
mean: 10.57
Density
ENO1 expression (tp10k)
D
Non-target knockdown
On-target knockdown
mean: 0.13
mean: 1.00
Density
Expression Relative to Control
E
mRNA Expression
sgRNA target
Expression relative to cells with control sgRNAs
F
Control sgRNA
Gapdh sgRNA
anti-Gapdh polyA mRNA
G
Gapdh-targeting sgRNA
Control sgRNA
Density
anti-Gapdh intensity (s.d.)
H
Significant Imaging Phenotype
significant n = 84
not significant n = 318
n = 3
not significant n = 47
Targeting sgRNAs
Control sgRNAs
I
Comparison of imaging and sequencing
log(imaging energy distance)
log(sequencing energy distance)
R = 0.5
J
Differential expression
Targeting sgRNA
Control sgRNA
Differentially Expressed Genes
K
Number of modulated channels
Targeting sgRNA
Control sgRNA
Differentially Intense Channels
