# Supplementary material for: Perturb-Multimodal: a platform for pooled genetic screens with sequencing and imaging in intact mammalian tissue
Source: Cell. Author manuscript; Available in PMC 2025 Aug 6. (PMC12324982; doi:10.1016/j.cell.2025.05.022)
Supplement: 8 — Table S1: Fluorescent readout oligonucleotide bits and RCA-MERFISH padlock probe sequences, related to Figures 1 and 3)○ The sequences and colors of the fluorescent readout oligonucleotide bits used for all RCA-MERFISH imaging.○ The targets and sequences of the padlock probes used for the mRNA measurements in RCA-MERFISH.○ The targets and sequences of the padlock probes used for the perturbation barcode measurements in RCA-MERFISH. [file NIHMS2091173-supplement-8.pdf]

Method S1

Related to Figures 1 and 3

Part A: Multiplexed immunostaining + RCA-MERFISH

Sample preparation occurs over a series of steps that take place either in reaction chambers or in wash dishes.

Reaction chambers are used for small-volume steps such as antibody staining, MelphaX treatment, padlock probe hybridization, and enzymatic ligation and rolling circle amplification. Reaction chambers are 10 cm dishes with a square of clean parafilm in the center. Reaction chambers are sprayed with RNaseZap and blotted very thoroughly with clean kimwipes until they are dry. 100 ul of reaction solution is added to the center of the parafilm square and the sample is inverted onto the drop of reaction solution.

Wash dishes are 6 cm tissue culture plates and are used with 5 ml of wash solution.

Part numbers for chemicals and enzymes are in the STAR Methods section.

Day 1

Step 1: Prepare slides

- Use silanized slides.
- Prepare 10% dilution of ThermoFisher poly-lysine solution in water.
- Put 100 ul drops of poly-lysine solution onto a piece of parafilm.
- Invert slides onto the drops and incubate for 30 minutes at RT.
- Wash slides 3x in water.
- Make 50 mL tube with 2X SSC + 3 drops of fiducial beads.
- Put slides into wash dishes and wash 3x with water.
- Dry slides very thoroughly in a 60˚ oven.

Step 2: Section tissue

- Set cryostat to -20oC. Set sectioning thickness to 10 um.

- Cut sections and air dry for 15 min at RT.
- Post-fix in 4% PFA for 15 min at RT.
- Wash 3x in PBS.
- Note: if conducting a fixed cell Perturb-seq experiment, also generate 100 um sections. Collect in an eppendorf or falcon tube and store at -80˚ until needed.

Step 3: De-crosslink

- Wash slides 3x with TE pH 9.
- Put in 60oC oven for 60 min.
- Cool to RT on bench.
- Wash 3x with PBS.

Step 4: Stain antibody-oligo conjugates

- Prepare antibody blocking buffer:
    - 615 ul water
    - 100 ul 10x PBS
    - 200 ul 50 mg/ml BSA
    - 30 ul 10% Triton X-100
    - 50 ul sheared salmon sperm DNA
    - 5 ul SUPERase·In RNase Inhibitor
- Place the slide on a piece of clean parafilm with the sample facing up. Add 250 ul of blocking buffer atop the sample. Incubate at RT for 30 minutes.
- Thaw and pool antibody-oligo conjugates. Dilute the antibody-oligo conjugates in the appropriate quantity of blocking buffer.
- Prepare a reaction chamber. Add 100 ul of the antibody-oligo/blocking solution to the parafilm in the reaction chamber.
- Take the sample and blot off the blocking buffer. Place the slide sample side-down on the antibody-oligo/blocking solution in the reaction chamber.
- Incubate at 4˚ for 16 hours.

Step 5: Wash and post-fix antibody-oligo conjugates

- Add PBS to a 6 cm tissue culture dish.
- Remove the slide from the reaction chamber and place face up in the PBS.
- Wash 4x in PBS and leave at RT in PBS for 15 minutes.

- Postfix with 4% PFA in PBS for 5 minutes.
- Wash 5x with PBS and incubate at RT for 20 minutes.
- Postfix with 1.5 mM BS(PEG)9 in PBS for 20 minutes, at RT.
- Incubate in PBS + 100 mM Tris pH 8 for 5 minutes.
- Wash 3x in PBS
- Note: at this stage, the sample can be stored at 4˚ in 70% EtOH in a parafilm-wrapped tissue culture dish for at least several weeks.

Step 4: Anchor RNA

- Prepare MelphaX mix (1:10 mixture of MelphaX stock + 20 mM MOPS pH 7.7).
    - MelphaX stock is prepared according to the recipe in Wang *et al*, 2021 (10.1016/j.cell.2021.11.024).
- Prepare a reaction chamber.
- Wash the sample 3x in MOPS pH 7.7
- Invert the sample face-down onto a 100 ul drop of MelphaX mix.
- Incubate at 37oC for 2 hrs.
- Wash 3x in PBS.

Step 5: Form gel + digest

- Prepare gel monomer solution:
    - 5 mL 40% acrylamide (19:1 acrylamide:bis-acrylamide)
    - 3 mL 5M NaCl
    - 3 mL 1M Tris
    - 100 ul TEMED
    - Water to 50 mL
- Degas for 15 min in a vacuum chamber.
- Prepare 10% APS solution in water (weigh X mg of APS, then resuspend in 10*X ul of H2O).
- Add 5 mL of gel mix to each slide. Incubate for 15 min at 4˚.
- Cool the gel mix and APS solution on ice.
- Clean a glass plate with RNaseZap and then with GelSlick. Place in a cold room.
- Take 1 mL of gel solution in a tube, on ice. Add 20 ul of murine RNase inhibitor + 20 ul 10% APS and mix thoroughly.
- Working in a cold room, put 50 ul drop of gel solution + APS on the glass plate.

- Blot off excess solution from the slide, then invert on top of the gel solution drop.
- Put a 50 g weight on top of the slide and push down to expel excess gel.
- Move to RT and let the gel form for 1.5 hours.
- Prepare digestion buffer:
    - 3 mL 20x SSC
    - 6 ml 10% SDS
    - 0.3 ml 25% Triton X-100
    - 1.5 mL 1M Tris pH 8
    - Water to 50 ml
- Gently remove the slide from the glass plate with a razor blade.
- Add 5 mL digestion buffer + 50 ul Proteinase K to a wash dish and wrap in parafilm.
- Digest at 47˚ for 24 hours.

Day 2

Step 5: Form gel + digest (continued)

- Replace the digestion buffer with fresh digestion buffer + 50 ul Proteinase K
- Digest at 47˚ for another 24 hours.

Day 3

Step 5: Form gel + digest (continued)

- Briefly wash with PBS + 0.1% Triton X-100 (PBSTx)
- Transfer slide to new dish, wash 3X with PBS + Triton X-100
- Wash slide 2x in 2X SSC and 2X in 2X SSC + 30% formamide. Equilibrate in 2X SSC + 30% formamide for at least 5 minutes.

Step 6: Hybridize library

- Prepare hyb mix (100 ul):
    - 10 ul 20X SSC
    - 30 ul formamide
    - 25 ul 20% PEG35K (final 5%)
    - 1 ul murine RNase inhibitor
    - 1 ul yeast tRNA
    - 10 ul RCA-MERFISH library (ideally at 1 nM/probe final concentration)

    - 1 ul 100 uM polyT anchor
    - 22 ul H2O
- Prepare a reaction chamber.
- Put 100 ul of hyb mix on parafilm.
- Take slide and remove excess liquid with a kimwipe. Invert face down onto the hyb mix.
- Add a water-saturated kimwipe to the reaction chamber to keep it humidified during hybridization.
- Incubate at 37˚ for 48 hours.

Day 5

Step 7: Wash + ligate

- Remove slide from incubator and place into a wash dish with 2X SSC + 30% formamide.
- Incubate at 47˚ for 30 minutes. Replace the 2x SSC + 30% formamide and incubate at 47˚ for 30 minutes a second time.
- Wash 3x in PBS.
- Prepare ligation mix on ice:
    - 10 ul SplintR ligase
    - 50 ul QuickLigase Buffer
    - 1 ul murine RNase inhibitor
    - 1 ul 100 uM RCA primer
    - 38 ul water
- Add 100 ul of ligation mix to a clean reaction chamber.
- Take the slide and remove excess liquid with a kimwipe.
- Invert face-down onto the ligation mix.
- Ligate at 37˚ for 2 hrs.

Step 8: RCA

- Wash slide 3x in PBS.
- Prepare RCA reaction mix on ice:
    - 10 ul Phi29 buffer
    - 10 ul BSA
    - 10 ul Phi29
    - 1 ul 25 mM dNTP

- 0.1 ul 50 mM aminoallyl-dUTP
- 1 ul murine RNase inhibitor
- 68 ul water

- Add RCA reaction mix to a clean reaction chamber.
- Take the slide and remove excess liquid with a kimwipe
- Invert face-down onto the RCA mix.
- Incubate at 4˚ for one hour.
- Incubate at 37˚ for two hours.
- Transfer to a dish with 5 mL of 1 mM BS(PEG)9. Postfix at RT for 30 minutes.
- Wash 3x in PBS.
- Store in PBS at 4˚ (stable for at least 1 month)

Part B: RCA Probe Amplification Protocol

Step 1: Twist PCR

- Prepare PCR master mix: 50 ul PCR, 36 PCRs per prep with 10% extra volume:
    - 970 ul H2O
    - 10 ul 100 uM forward primer
    - 10 ul 100 uM reverse primer
    - 10 ul Twist library (resuspended at 1 ng/reaction)
    - 1000 ul 2X Phusion master mix
- [Optional: qPCR If don't know number of cycles]
    - Take 1 50 ul reaction
    - Run on qPCR machine using appropriate Tm for primers and per-cycle protocol below to determine the number of cycles required to reach the mid-exponential phase.
- PCR:
    - Aliquot PCR master mix into 36 wells of 96 well plate.
    - PCR protocol:
        - 98oC for 30 s
        - Repeat N times:
            - 98oC for
            - 66-69oC depending on primer
            - 72oC
- Purify PCR products with SPRISelect beads:
    - Pool PCR products in 5 mL tube.
    - Add 1.2X volume of SPRISelect beads.
    - Mix thoroughly.
    - Incubate for 5 min at RT.
    - Bind beads to magnet.
    - Remove supernatant.
    - Wash with 80% EtOH for 1 min.
    - Remove EtOH.
    - Wash with 80% EtOH for 1 min.
    - Remove EtOH as thoroughly as possible.
    - Remove from magnet.
    - Resuspend beads in 200 ul water.

- Elute for 5 min at RT.
- Put on magnet, allow beads to bind.
- Transfer supernatant to 1.5 mL Eppendorf tube
- *Expected result: ~100 ng/ul DNA for 500 gene library with 20 PCR cycles*

Step 2: Ligation

- Goal is to blunt end ligate PCR products so that some become circularized
- Set up ligation in 1.5 mL Eppendorf tube:
  - 170 ul DNA = final conc of ~4-8 ng/ul
  - 500 ul QL Buffer
  - 50 ul QL
  - 280 ul H2O
- Ligate overnight at room temperature.
- Purify with 1.2X SPRISelect as described in “Purify PCR products with SPRISelect beads” above:
  - Use 1200 ul SPRISelect beads + 1000 ul ligation
  - Elute in 110 ul water

Step 3: Nicking

- Goal is to nick DNA for RCA amplification.
- Set up nicking reaction:
  - 100 ul DNA
  - 10 ul Nt.BbvCI
  - 15 ul CutSmart
  - 25 ul H2O
- Split into 3 50 ul reactions in PCR strip.
- Run on PCR machine:
  - 37oC for 1 hour
  - 80oC for 20 min

Step 4: RCA reaction

- Pool nicking product (total volume 180 ul)
- Do 3X bead cleanup as described above, eluting in 180 ul water

- Make RCA master mix in 5 mL tube (48 50 ul reactions = 2.4 mL total):
    - 240 ul 10X Phi29 Buffer
    - 120 ul Phi29
    - 120 ul 10 mM dNTPs
    - 15 ul of T4 gene 32 + 48 ul of ET SSB
    - 180 ul DNA
    - 1692 ul H_2O
- Aliquot into 50 ul reactions in 96 well plate.
- Incubate at 30oC for 16-24 hours.
- Heat inactivate at 65oC for 10 min.
- Pool RCA products.
- Ethanol precipitate DNA:
    - Add 0.1X volume 3M sodium acetate + 3X volume ice cold 100% EtOH
    - Keep on ice for 1 hour.
    - Spin at max speed for 30 min in a chilled microcentrifuge.
    - Remove supernatant.
    - Resuspend pellet in ice-cold 70% EtOH to wash.
    - Spin at max speed for 10 min to re-pellet.
    - Resuspend pellet in cold 70% EtOH for a second wash.
    - Spin at max speed for 10 min to re-pellet.
    - Resuspend pellet in 800 ul of EB (10 mM Tris)
    - Note: liquid may be *extremely* viscous after resuspension if the RCA worked; resuspension may require heating.

Step 5: RCA product digestion

- Remove 20 ul of RCA product for QC.
- Add the following:
    - 30 ul 1mM BccI_short primer
    - 30 ul 1mM BciVI_short primer
    - 100 ul CutSmart
    - 10 ul H_2O
- Vortex and mix vigorously.
- Heat up RCA product + digestion primers to 95oC for 10 min in 1.5 mL Eppendorf tube.
- Cool to room temperature on the bench.

- Add restriction enzymes:
  - o 15 ul BccI
  - o 15 ul BciVI
- Mix thoroughly.
- Incubate at 37oC O/N.
- Purify products with Jeff Moffitt's Purification beads (Encoding Probe Construction Protocol, Moffitt Laboratory, 2021; Moffitt Lab Github):
  - o Pool PCR products in 5 mL tube.
  - o Add 3X volume of beads (3 mL)
  - o Mix thoroughly.
  - o Let incubate for 15 min at RT.
  - o Bind beads to magnet.
  - o Remove supernatant.
  - o Wash with 70% EtOH for 1 min.
  - o Remove EtOH.
  - o Wash with 70% EtOH for 1 min.
  - o Remove EtOH as thoroughly as possible.
  - o Remove from magnet.
  - o Resuspend beads in 400 ul TE buffer.
  - o Let sit for 15 min at RT.
  - o Put on magnet, allow beads to bind.
  - o Remove supernatant to 1.5 mL tube.

QC:

- Digest 10 ul of RCA product *without* annealed primers for 1 hr at 37oC with 1 ul of Bcci and 1 ul of BciVI
- Analyze PCR product, ligated product, and final digestion product on a denaturing PAGE gel.
